# Data Driven Explanation of Temporal and Spatial Variability of Maize Yield in the United States

**DOI:** 10.3389/fpls.2021.701192

**Published:** 2021-09-21

**Authors:** Lizhi Wang

**Affiliations:** Department of Industrial and Manufacturing Systems Engineering, Iowa State University, Ames, IA, United States

**Keywords:** crop yield prediction, machine learning, crop models, temporal and spatial variability, heuristic algorithm

## Abstract

Maize yield has demonstrated significant variability both temporally and spatially. Numerous models have been presented to explain such variability in crop yield using data from multiple sources with varying temporal and spatial resolutions. Some of these models are data driven, which focus on approximating the complex relationship between explanatory variables and crop yield from massive data sets. Others are knowledge driven, which focus on integrating scientific understanding of crop growth mechanism in the modeling structure. We propose a new model that leverages the computational efficiency and prediction accuracy of data driven models and incorporates agronomic insights from knowledge driven models. Referred to as the GEM model, this model estimates three independent components of (G)enetics, (E)nvironment, and (M)anagement, the product of which is used as the predicted crop yield. The aim of this study is to produce not only accurate crop yield predictions but also insightful explanations of temporal and spatial variability with respect to weather, soil, and management variables. Computational experiments were conducted on a data set that includes maize yield, weather, soil, and management data covering 2,649 counties in the U.S. from 1980 to 2019. Results suggested that the GEM model is able to achieve a comparable prediction performance with state-of-the-art machine learning models and produce meaningful insights such as the estimated growth potential, effectiveness of management practices, and genetic progress.

## 1. Introduction

Crop yield prediction plays an important role in agriculture. On the economic front, agriculture stakeholders such as farmers, insurance companies, and breeders rely on yield predictions to make informed operational decisions. On the societal front, yield predictions help governments and organizations make effective policies to strength global security, support famine-prevention efforts, and protect environmental sustainability, especially in an era of global climate change and pandemics (Messina et al., 2010; Marko et al., 2016). On the scientific front, underlying crop yield prediction is a fundamental research question of understanding how phenotype is determined by genotype, environment, and their interactions. In particular, the relationship between genetics, weather, soil, and management variables and crop yield has been the topic of extensive studies. The pursuit of more accurate crop yield prediction techniques has and will continue to motivate innovations at the intersection of plant science, engineering, and data analytics.

Most crop yield prediction models can be categorized as either data driven or knowledge driven. Machine learning models, epitomized by neural networks, consist of large numbers of simple computational units that grow into complex model structures for data driven analysis. With little pre-programmed knowledge or biases, a machine learning algorithm treats crop yield as an unknown function of genotype and environment and attempts to approximate the underlying function by learning its own lesson from large data sets. Khaki and Wang (2019) used a deep neural network model for the 2018 Syngenta crop challenge (Syngenta, 2020), in which participants were challenged to predict the 2017 crop yield of 2,247 fields using historical data of genotype, weather, soil, and yield. Their approach outperformed other popular machine learning methods such as LASSO, shallow neural networks, and regression tree. Shahhosseini et al. (2019) evaluated four machine learning algorithms and their ensembles in predicting maize yield and nitrate losses. Using experimental data from seven locations in four Midwestern states in the U.S. over 5–7 years and a large scenario analysis data set generated by the agricultural production systems simulator (APSIM) (Holzworth et al., 2014), they achieved the following RMSEs in bu/ac for yield prediction: 22.19 for LASSO regression, 22.42 for ridge regression, 20.82 for random forests, 20.04 for extreme gradient boosting, and 18.44 for their optimal ensemble. Kang et al. (2020) compared six machine learning algorithms in predicting county level maize yield in 12 states in the U.S. using data from 2001 to 2016. The RMSE ranged from 14.8 to 24 bu/ac. Crane-Droesch (Crane-Droesch, 2018) proposed a semiparametric variant of a deep neural network and compared it with multiple other machine learning algorithms using data from 9 states in the U.S. Corn Belt from 1979 to 2016; RMSEs for unseen years ranged from 15.9 to 19.1 bu/ac. More detailed reviews of machine learning models for crop yield prediction can be found in Chlingaryan et al. (2018) and van Klompenburg et al. (2020).

Knowledge driven models, epitomized by crop models such as APSIM (Holzworth et al., 2014) and CERES-Maize (Hodges et al., 1987), build upon physiological understanding of plant growth processes and develop biologically meaningful non-linear equations to predict crop yield (among other outputs) as a complex function of plant traits (e.g., leaf appearance rate, total leaf number, grain fill duration, grain number, and root front velocity) and environmental parameters (Batchelor et al., 2002; Heslot et al., 2014; Schauberger et al., 2017). Crop models offer biological insights into causes of phenotypic variability by providing explicit explanations of the interactions between traits and environmental conditions in different phases of the crop growth cycle. As such, knowledge driven models are more commonly evaluated based on their qualitative reflection of crop responses to agrometeorological effects (Lalić et al., 2014) than quantitative prediction accuracy (Kiniry et al., 1997). Blanc (2017) built an emulators of crop yields based on an ensemble of five crop models and evaluated its performance in replicating spatial patterns of yields crop levels and changes overtime. Schauberger et al. (2017) used an ensemble of nine crop models to enhance the ability of individual process-based crop models to represent effects of high temperature on crop yield. Durand et al. (2018) assessed the ability of 21 crop models to capture the impact of elevated carbon dioxide concentration on maize yield and found evidence that more mechanistic modeling approaches led to better performances.

Data driven and knowledge driven models have complementary strengths and limitations. On the one hand, the ability to approximate complex functions to fit data (Hornik et al., 1990) enables machine learning models to achieve relatively high prediction accuracy, but a major limitation is the difficulty to explain the results. For example, many studies use a separate model for each geographic region with different parameters; each feature is used in hundreds or thousands of equations to produce the final prediction, and the importance of different features changes by year and by location (Kang et al., 2020). As a result, when the predictions are accurate, they offer little insights that are explainable, much less transferable temporally or spatially; when the predictions are inaccurate, it is hard to identify the cause of the errors. On the other hand, knowledge driven models have the potential to propose scientifically and biologically meaningful hypotheses that can form the basis of experimental validation. The pre-programmed human input in knowledge driven models can greatly simplify the learning process while achieving a reasonable performance, but it also restricts what can be learned from data and consequently limits the prediction accuracy. Parameter calibration is also challenging due to the complex structures of these models.

Attempts have been made to integrate more human knowledge in data driven models. Khaki et al. (2020) designed a novel machine learning model that uses convolutional neural networks to extract interactions between weather and soil variables and recurrent neural networks to capture time dependencies of genetic improvement of seeds. Using data from 13 states in the U.S. Corn Belt, with 1980 to 2015 being training years, the model achieved RMSEs of 16.48, 15.74, and 17.64 bu/ac for the respective test years of 2016, 2017, and 2018. Coupled with the backpropagation method, the model could reveal the extent to which weather conditions, accuracy of weather predictions, soil conditions, and management practices were able to explain the variability in the crop yields. Several other studies have used regression models with manually extracted features to estimate effects of temperature (Zhao et al., 2017; Butler et al., 2018; Tigchelaar et al., 2018), solar brightening (Tollenaar et al., 2017), plant density (Lacasa et al., 2020), and flowering time (Parent et al., 2018) on crop yield.

We propose a new model for not only predicting crop yield but also attributing spatial and temporal yield variability to contributions of genetics, environment, and management variables. This is also a largely data driven model, but the model structure was specifically designed to incorporate basic agronomic knowledge to ensure that parameters from the trained model can be used to explain temporal and spatial yield variability with respect to genetic, environment, and management components. In contrast, parameters from most machine learning models are typically much more numerous and harder to provide meaningful interpretations beyond predicted yield. A large set of weather, soil, and management data were collected from public sources, which covered 41 states in the U.S. from 1980 to 2019. The model was designed for this data set in order to strike a balance among four competing objectives. First, modeling resolution takes full advantage of available data to accurately quantify the effects of daily weather changes and variability in soil conditions and management practices on crop yield. Second, modeling structure is based on scientific facts or reasonable simplifying assumptions. Third, modeling results can be used to explain causes of temporal and spatial yield variability, allowing users to either gain meaningful insights or pinpoint flaws in specific model components for further improvement. Fourth, modeling parameters can be optimally calibrated using state-of-the-art machine learning and optimization techniques to extract useful information from data that is transferable temporally and spatially. We focused on maize yield prediction because maize is one of the most important food, feed, and fuel crops in the U.S., and its production has demonstrated temporal and spatial variability and strong sensitivity to both environmental and management conditions (Meng et al., 2016).

## 2. Data

This section provides details on the data we collected for this study. More detailed weather, soil, or management data that were only available at smaller temporal and spatial scales were not included in this study. Moreover, we were not able to locate publicly accessible data sources for seed genetics. Instead, we used modeling techniques to estimate the genetics component in crop yield based on available data sets. This was achieved by using a polynomial function to explain part of the historical yield that could not be explained by spatial or temporal variability in soil and weather conditions. More details about the modeling approach can be found in section 3.

### 2.1. Yield and Geography Data

**Figure 1:**
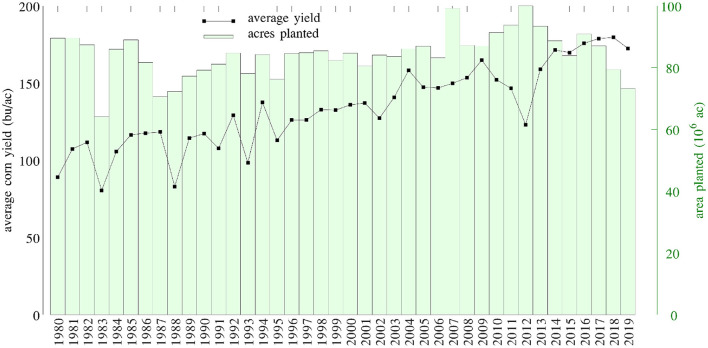
National average corn yield and areas planted from 1980 to 2019.

**Figure 2:**
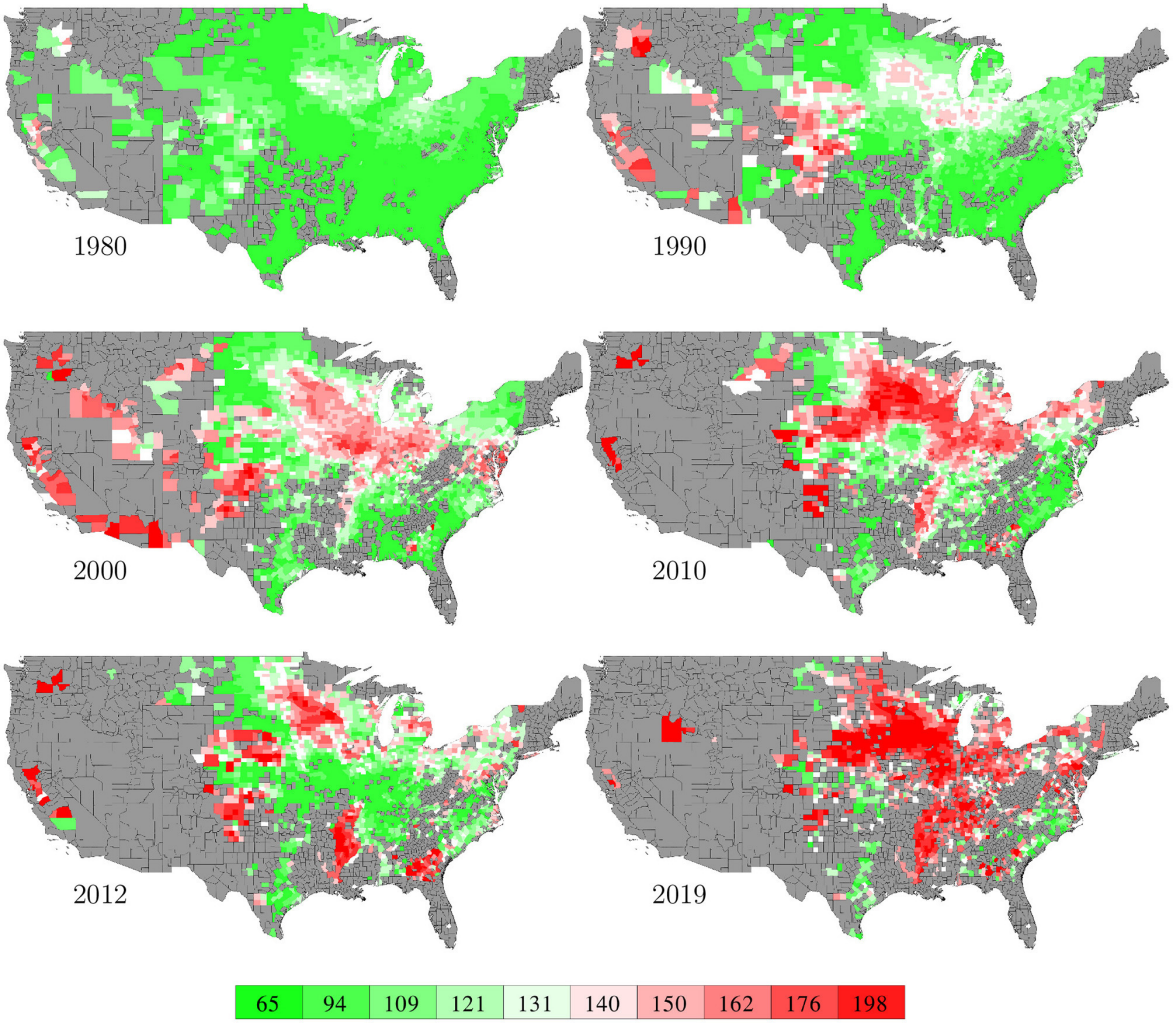
County level average corn yield in 6 representative years. The color map shows the averages of the 0–10th, 10–20th,…, 90–100th percentile intervals of all county-year combinations from 1980 to 2019. Counties without yield data are shown in gray.

County level corn yield in the U.S. from 1980 to 2019 were collected from NASS (2020). After removing a few data points with incomplete information, the entire data set contained 2,649 counties in 295 crop reporting districts of 41 states (all 50 states in the U.S. excluding AK, CT, HI, MA, ME, NV, NH, RI, and VT; the list of the 41 states can be found in the horizontal axis of **Figure 19**) with a total of 78,169 corn yield records for county-year combinations. Figure 1 shows the temporal trend of national average corn yield (as well as areas planted) from 1980 to 2019. Figure 2 visualizes the spatial variability of county level average corn yield in 6 representative years: 1980, 1990, 2000, 2010, and 2019 in approximate 10-year time lapse plus 2012, in which year severe drought resulted in historic reductions in corn yield.

Shape files of U.S. counties were collected from National Weather Service (2020). This information was used to determine the membership of counties in crop reporting districts and states and also to locate weather stations and soil map units for calculating average weather and soil variables within each county.

### 2.2. Weather Data

Daily surface weather data on a 1-km grid from 1980 to 2019 were collected from Daymet (Thornton et al., 2020). The data set included 7 variables, the names and descriptions of which from Thornton et al. (2020) are summarized as follows.

- dayl: duration of the daylight period in seconds per day.
- prcp: daily total precipitation in millimeters per day, sum of all forms converted to water-equivalent.
- srad: incident shortwave radiation flux density in watts per square meter, taken as an average over the daylight period of the day.
- swe: snow water equivalent in kilograms per square meter.
- tmax: daily maximum 2-meter air temperature in degrees Celsius.
- tmin: daily minimum 2-meter air temperature in degrees Celsius.
- vp: water vapor pressure in pascals.

**Figure 3:**
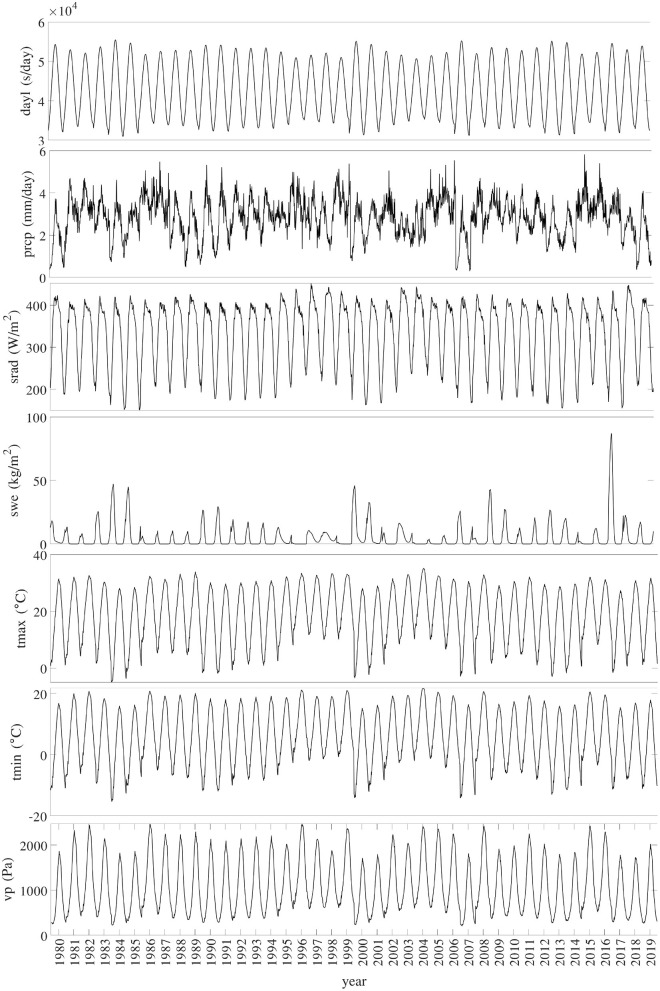
Trend of national averages of 7 weather variables from 1980 to 2019.

**Figure 4:**
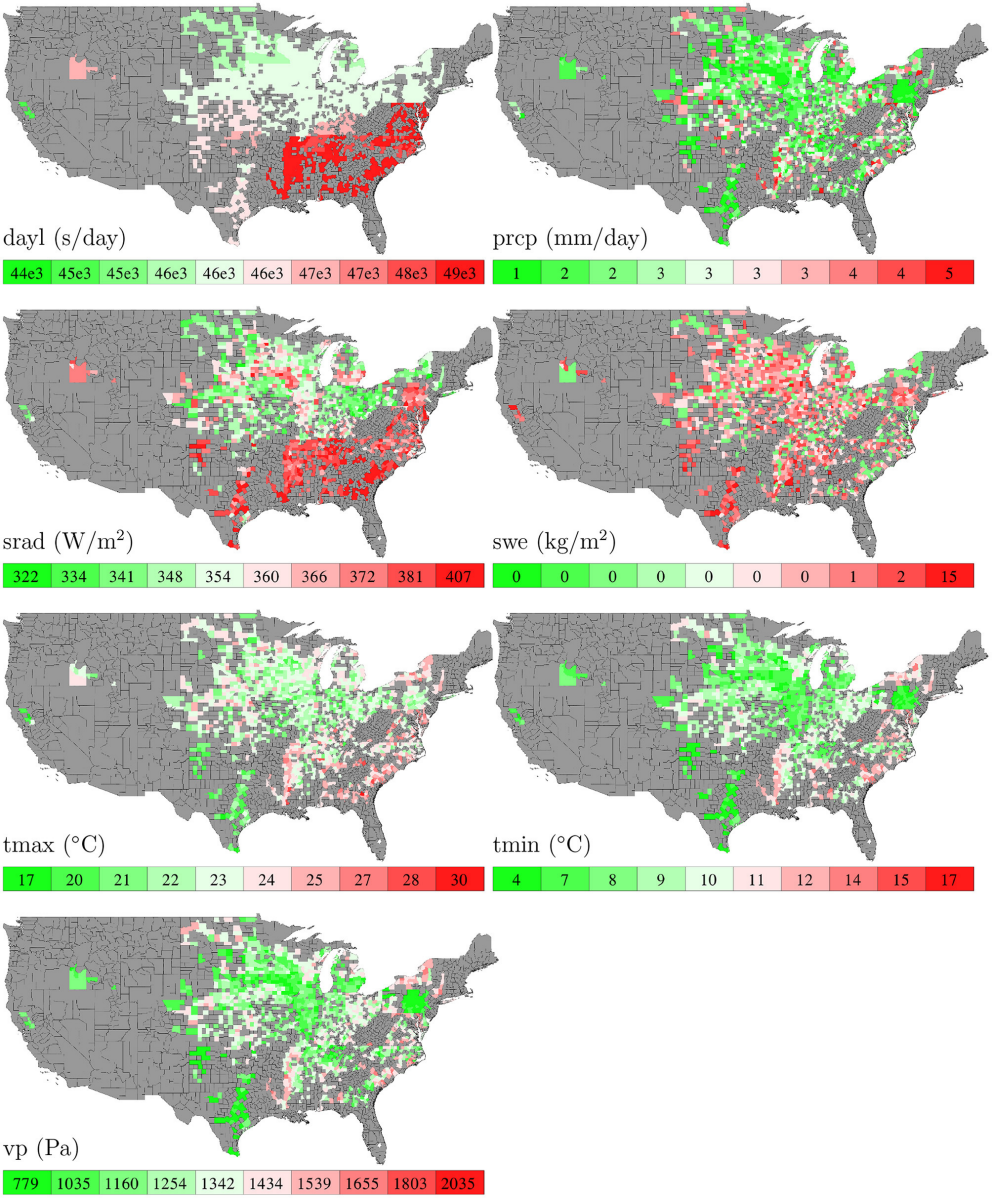
County averages of 7 weather variables between planting and harvesting weeks in 2019. The color map shows the averages of the 0–10th, 10–20th,…, 90–100th percentile intervals of all county-year combinations from 1980 to 2019. Although weather data are available for all counties in all years, those without yield data are shown in gray.

Figure 3 shows the temporal trends of national averages of the 7 weather variables from 1980 to 2019. Figure 4 shows the spatial variability of these weather variables between planting and harvesting weeks in 2019.

### 2.3. Soil Data

Soil data were collected from the latest version of Gridded Soil Survey Geographic (gSSURGO) Database released in July 2020 (USDA, 2020). We used 10 soil variables measured in the Value Added Look Up Table (Valu1) in the database, the names and descriptions of which from USDA (2020) are summarized as follows.

- aws: available water storage, expressed in mm, the volume of plant available water that the soil can store in this layer based on all map unit components. This variable was measured at 11 standard depth layers: standard zone 1 (0–5 cm depth), layer 2 (5–20 cm depth), layer 3 (20–50 cm depth), layer 4 (50–100 cm depth), layer 5 (100–150 cm depth), layer 6 (150 cm to the reported depth of the soil profile), zone 2 (0–20 cm depth), zone 3 (0–30 cm depth), zone 4 (0–100 cm depth), zone 5 (0–150 cm depth), and total soil profile (0 cm to the reported depth of the soil profile).
- tka: thickness of soil components, expressed in cm for the available water storage calculation. This variable was measured at 11 standard depth layers.
- soc: soil organic carbon stock estimate, expressed in grams C per square meter. This variable was measured at 11 standard depth layers.
- tks: thickness of soil components, expressed in cm for the soil organic carbon calculation. This variable was measured at 11 standard depth layers.
- nccpi3corn: National Commodity Crop Productivity Index for Corn (weighted average). Values range from 0.01 (low productivity) to 0.99 (high productivity).
- pctearthmc: National Commodity Crop Productivity Index for major earthy components, which are those soil series or higher level taxa components that can support crop growth.
- rootznemc: Root zone depth, expressed in mm, is the depth within the soil profile that commodity crop roots can effectively extract water and nutrients for growth.
- rootznaws: Root zone available water storage estimate, expressed in mm, is the volume of plant available water that the soil can store within the root zone based on all map unit earthy major components.
- droughty: Drought vulnerable landscapes comprise those map units that available water storage within the root zone for commodity crops is less than or equal to 6 inches (152 mm), expressed as “1” for a drought vulnerable soil landscape map unit or “0” for a non-droughty soil landscape map unit.
- pwsl1pomu: Potential Wetland Soil Landscapes is expressed as the percentage of the map unit that meets the PWSL criteria.

**Figure 5:**
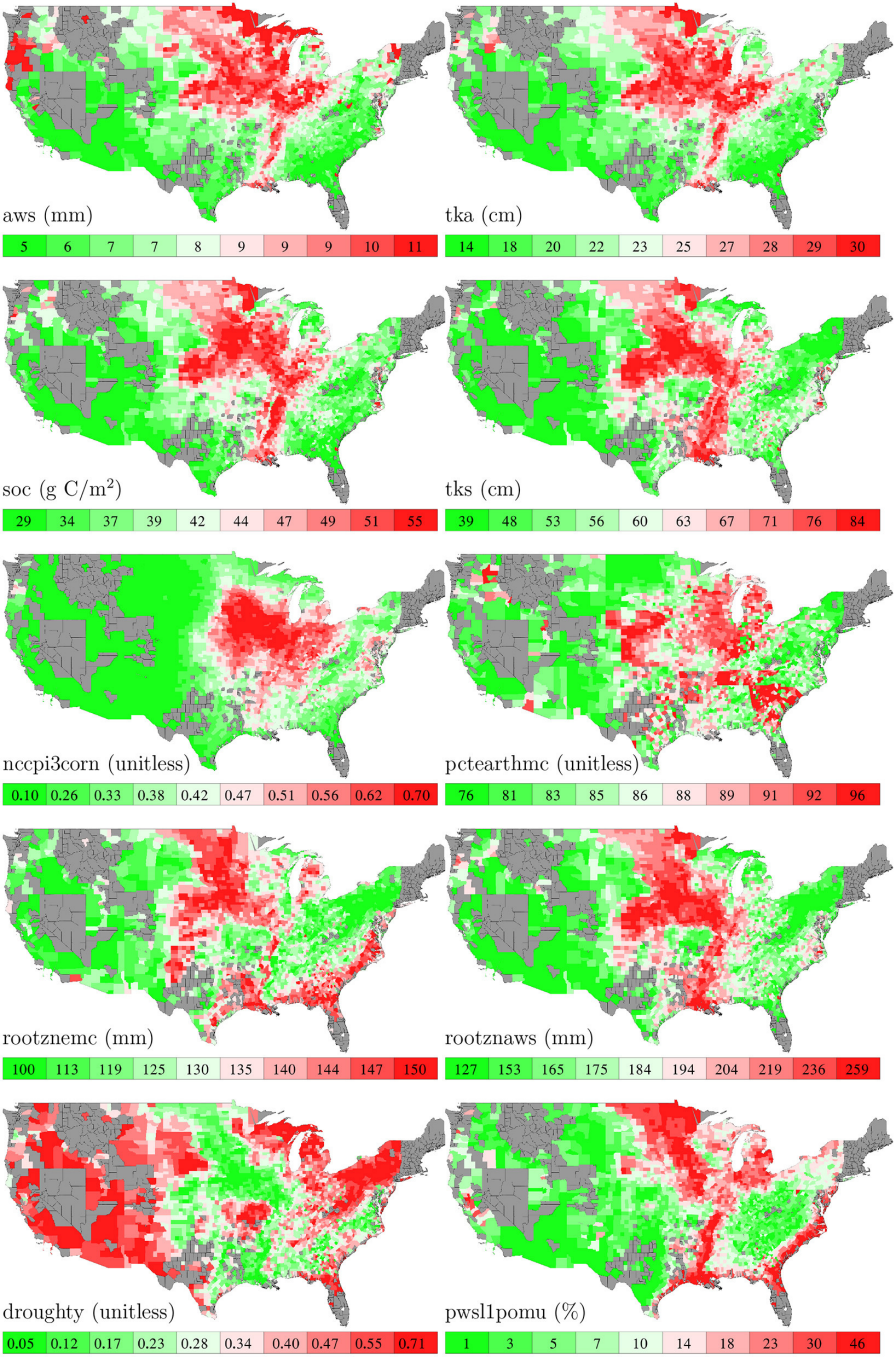
County averages of 10 soil variables. The color map shows the averages of the 0–10th, 10–20th,…, 90–100th percentile intervals of all county-year combinations. Although soil data are available for all counties, those without yield data are shown in gray.

Figure 5 shows the spatial variability of these 10 soil variables.

### 2.4. Management Data

**Figure 6:**
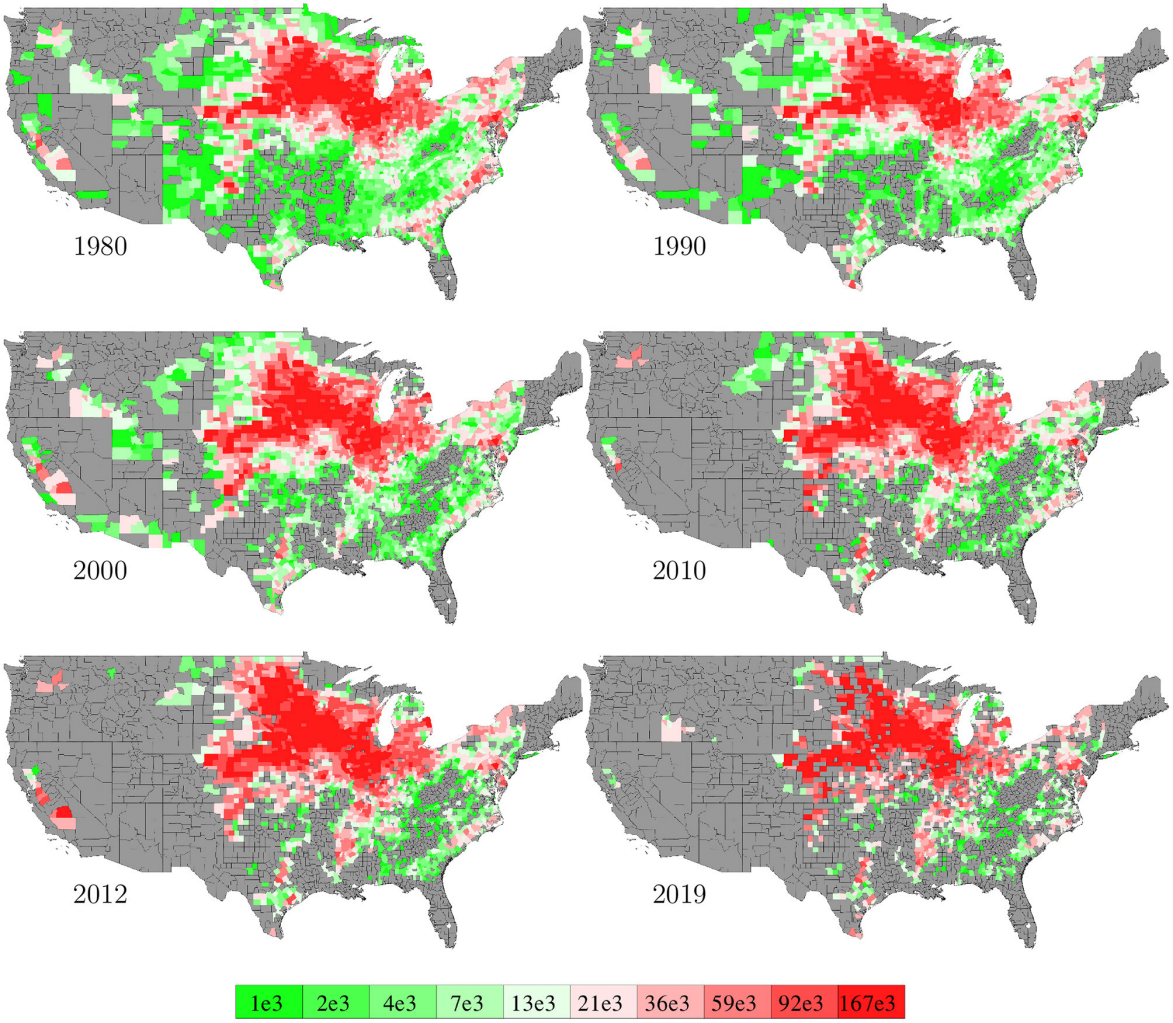
County averages of areas planted in 6 representative years. The color map shows the averages of the 0–10th, 10–20th,…, 90–100th percentile intervals of all county-year combinations. Counties without such data are shown in gray.

Data for areas planted in the 2,649 counties in the U.S. from 1980 to 2019 were collected from NASS (2020). The temporal trend of this information was shown in Figure 1 together with the yield trend. Figure 6 visualizes the spatial variability of areas planted in 6 representative years. Due to limited data availability, several important management variables were not included in the model, such as seed genotype, irrigation, fertilization, tillage, and disease/weed control.

**Figure 7:**
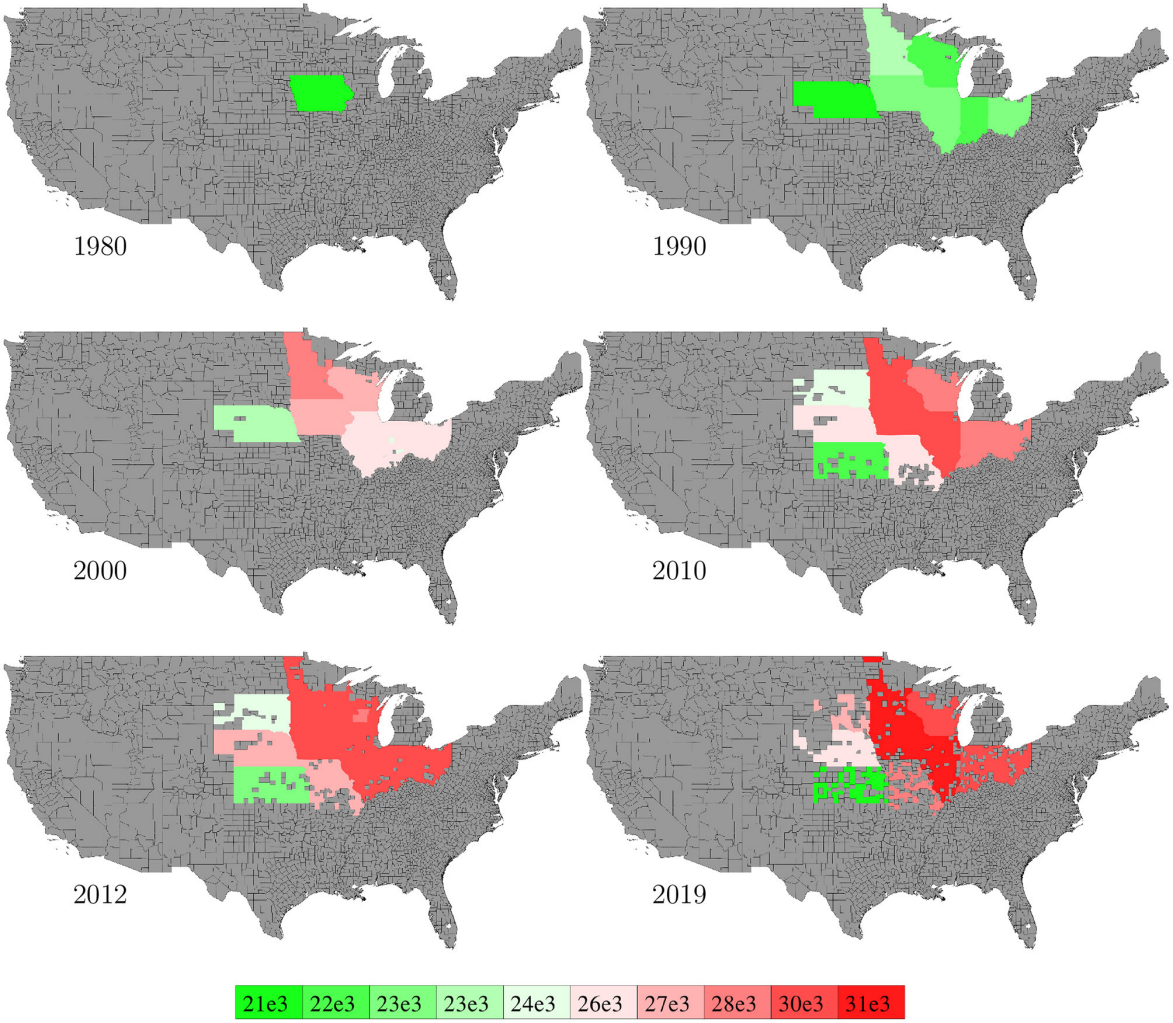
State level population density in 6 representative years. The color map shows the averages of the 0–10th, 10–20th,…, 90–100th percentile intervals of all state-year combinations. States without such data are shown in gray.

Data for corn plant population density (number of plants per acre) in the U.S. from 1980 to 2019 were collected from NASS (2020). Data were available at the state level with more than 60% missing values, and we used mean of non-missing data (other years for the same state, if available) for data imputation. Figure 7 visualizes the spatial variability of plant density in 6 representative years. Imputed data were not shown in the figure.

**Figure 8:**
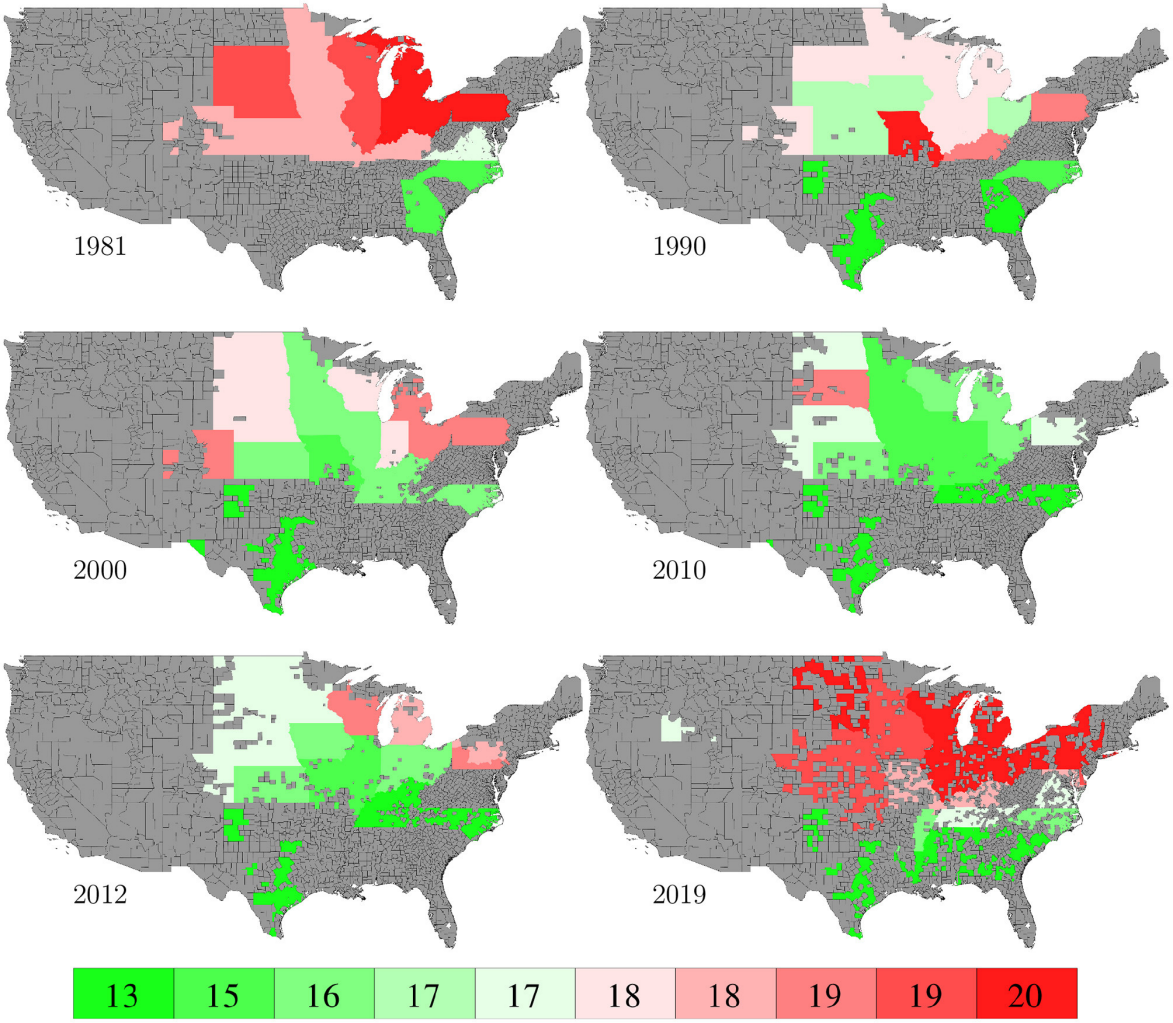
Week numbers when 50% planting was finished in 6 representative years. The color map shows the averages of the 0–10th, 10–20th,…, 90–100th percentile intervals of all state-year combinations. States without such data are shown in gray.

**Figure 9:**
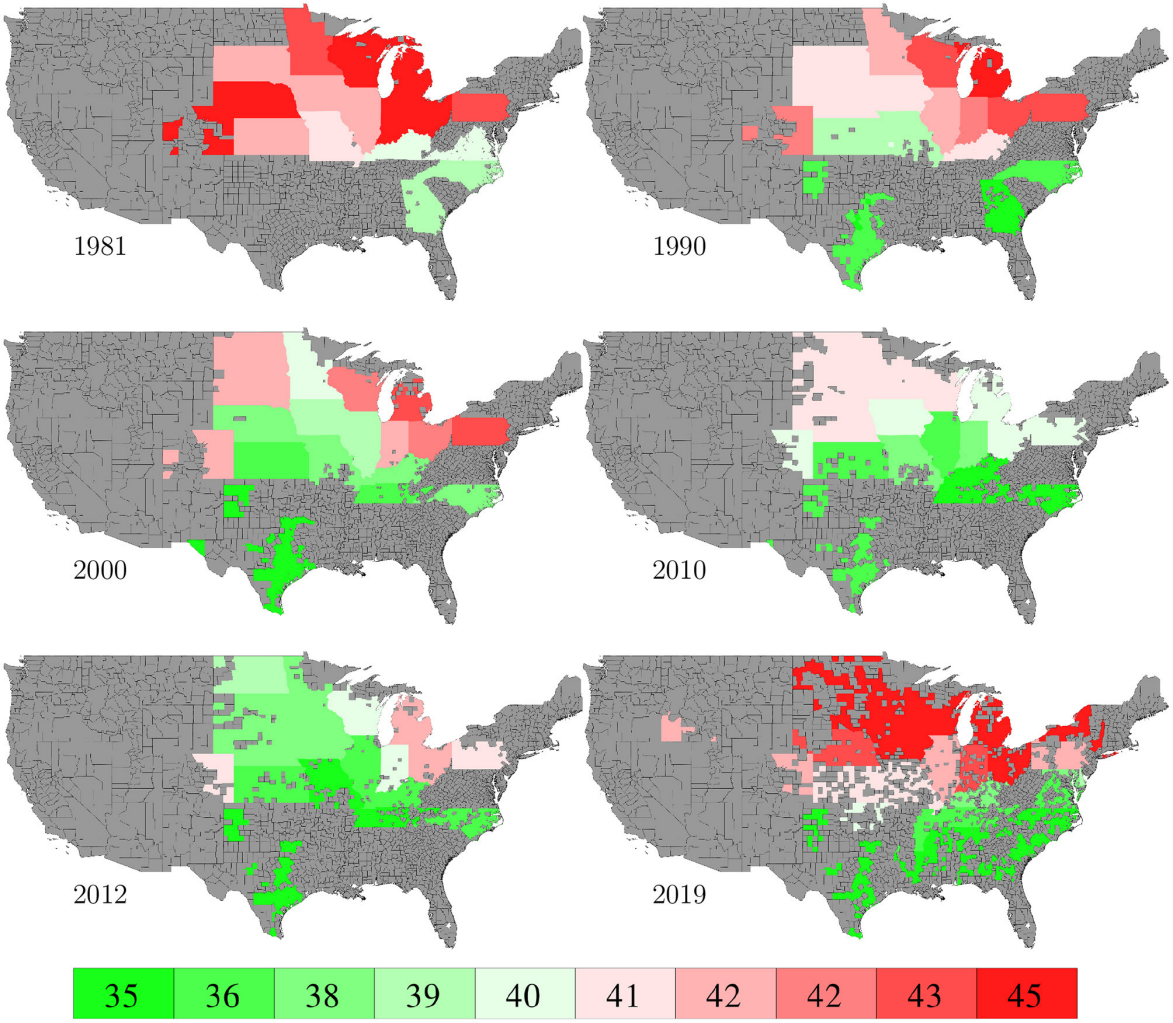
Week numbers when 50% harvesting was finished in 6 representative years. The color map shows the averages of the 0–10th, 10–20th,…, 90–100th percentile intervals of all state-year combinations. States without such data are shown in gray.

Planting and harvesting time data from 1980 to 2019 were collected from NASS (2020), which were at the state level given as percentages of planted areas having finished planting or harvesting in each week. Although 30% data were missing, the format of the planting and harvesting time data allowed imputation to be done in a relatively straightforward manner using the mean of non-missing data. Fortunately, the planted areas of corn belt states with more complete data far exceeded other states with more missing data, as shown in **Figure 19**, which limited the negative impact of the missing data on this case study. Figures 8, 9 visualize the spatial variability of planting and harvesting times in 6 representative years (1980 was replaced with 1981 because no harvesting data were available for 1980).

## 3. Model

The model was designed for explaining temporal and spatial variability of corn yield in the U.S. using available data summarized in section 2. Compared with existing crop yield prediction models, this model has three salient features. First, it integrates domain knowledge of plant science in the design of the model. Second, it deploys advanced machine learning and optimization algorithms as solution techniques. Third, it was designed to make data-driven discoveries on the interactions among genetics, environment, and management variables, which must be validated in 2,649 counties in 41 states over the past 40 years. We refer to this model as the GEM model, due to its capability to dissect and quantify (G)enetics, (E)nvironment, and (M)anagement components of crop yield.

### 3.1. Assumptions

**Assumption 1:** Crop yield is jointly determined by three mutually independent components: genetics, environment, and management. The environment component, determined by weather and soil variables and their interactions, sets a growth potential. The genetics and management components reflect the proportion of growth potential that is actually captured and converted to crop yield.

**Assumption 2:** The genetics component within a crop reporting district is the same for each year. This assumption enables the model to capture the effects of seed selections in different geographic regions; it also gives each crop reporting district sufficient data to learn a separate model for its own genetics trend. The change of genetic performance over the previous year is within a subjectively estimated range of [−2.5%, 5%].

**Assumption 3:** Environmental and management effects on crop growths during different time periods are additive. As such, the model calculates the amount of actual growth in each week based on G, E, and M variables of a given county, and the sum of the growths over 52 weeks gives the total yield for that county. Similar assumptions are commonly made when analyzing the effect of environmental variables. For example, growing degree days and killing degree days are used to measure the cumulative beneficial and damaging effects, respectively, of thermal time (Butler et al., 2018).

**Assumption 4:** The amount of crop growth potential achieved by management practices depends on the growth stages of the crop. For simplicity, we consider only two stages: the vegetative and reproductive stages, the division for which is approximated as the first week when 100% planting has been finished in a given county.

**Assumption 5:** Up to quadratic additive effects and bilinear interactions between weather and soil variables are considered. This assumption makes the model formulation relatively simple for computational efficiency yet sufficiently flexible for data-driven discovery.

### 3.2. Nomenclature

**Known parameters:**

- I: set of indices for 7 weather variables in the weather data set.
- J: set of indices for 10 soil variables in the soil data set.
- C: set of indices for 2,649 counties in the corn yield data set.
- R(c): set of indices for all counties that belong to the same crop reporting district as county *c*.
- T: set of years 1980 to 2019.
- CT: set of 78,169 county-year combinations for which historical corn yield data are available in the collected data set.
- W: set of 52 weeks in a year.
- $W^{\text{V}}\left(c,t\right)$: subset of 52 weeks in year t∈T that crop in county c∈C is in or before the vegetative stage.
- $W^{\text{R}}\left(c,t\right)$: subset of 52 weeks in year t∈T that crop in county c∈C is in or after the reproductive stage.
- *K*: the highest polynomial order of genetic progress as a function of time. *K* = 10 was used in this study.
- *A*_*c,t*_: area planted in county c∈C and year t∈T.

**Explanatory variables:**

- t∈T: year variable.
- *W*_*c,i,w*_: weather variable i∈I in county c∈C and week w∈W. This variable is averaged over 7 days and across all weather stations within county *c*. In case no weather stations were located inside a small county, the nearest one was used.
- *S*_*c,j*_: soil variable j∈J in county c∈C. This variable is averaged across all soil map units within county *c*. In case no soil map units were located inside a small county, the nearest one was used.
- *D*_*c,t*_: population density (number of plants per acre) in county c∈C and year t∈T.
- *P*_*c,w*_: percentage of planting finished in county c∈C by week w∈W, which monotonically increases from 0 to 100% during the planting season and stays at 100% to the end of the year.
- *H*_*c,w*_: percentage of harvesting finished in county c∈C by week w∈W, which monotonically increases from 0 to 100% during the harvesting season and stays at 100% to the end of the year.

**Response variables:**

- ŷ_*c,t*_: predicted corn yield in county c∈C and year t∈T.
- *y*_*c,t*_: observed corn yield in county c∈C and year t∈T.

**Unknown parameters:**

- α_*c,k*_: genetic progress parameter for *t*^*k*^ in county c∈C.
- $\gamma_{i,j}^{\text{V}}$: parameter of interaction between (weather and/or soil) variables i∈IorJ and j∈IorJ on growth potential during the vegetative stage. In particular, $\gamma_{0,0}^{\text{V}}$ is a constant term, $\gamma_{i,0}^{\text{V}}$ is the coefficient for linear effect of variable *i*, $\gamma_{i,i}^{\text{V}}$ is the coefficient for quadratic effect of variable *i*, and $\gamma_{i,j}^{\text{V}}$ is the coefficient for bilinear interaction between variables *i* and *j*.
- $\gamma_{i,j}^{\text{R}}$: parameter of interaction between variables *i* and *j* on growth potential during the reproductive stage.

### 3.3. Crop Yield Model

The GEM model predicts the corn yield in county *c* and year *t* as follows:


(1) $$\begin{array}{l} {ŷ}_{c,t}=\left(\overset{K}{\underset{k=0}{\sum }}\alpha_{c,k}t^{k}\right)\cdot \left[\underset{w\in W\left(c,t\right)}{\sum }D_{c,t}\left(P_{c,w}-H_{c,w}\right)G_{c,w}\right]. \end{array}$$

The term $\overset{K}{\underset{k=0}{\sum }}\alpha_{c,k}t^{k}$ estimates the relative genetic performance of seeds in county *c* and year *t* using a polynomial function of the year number (normalized to [0, 1]). The term *D*_*c,t*_(*P*_*c,w*_ − *H*_*c,w*_) reflects management practices of plant population density and planting/harvesting progress, which directly affect the amount of growth potential that can be captured and converted to grain yield. In particular, (*P*_*c,w*_ − *H*_*c,w*_) calculates the percentage of crop in county *c* and week *w* that has been planted and not yet harvested, as the crop continues to accumulate growth. The composite variable *G*_*c,w*_ calculates the growth potential in county *c* and week *w*, defined as $G_{c,w}=\gamma_{0,0}^{\text{V}}+\underset{i\in I}{\sum }\gamma_{0,i}^{\text{V}}W_{c,i,w}+\underset{i_{1}\leq i_{2}\in I}{\sum }\gamma_{i_{1},i_{2}}^{\text{V}}W_{c,i_{1},w}W_{c,i_{2},w}+\underset{j\in J}{\sum }\gamma_{0,j}^{\text{V}}S_{c,j}+\underset{j_{1}\leq j_{2}\in J}{\sum }\gamma_{j_{1},j_{2}}^{\text{V}}S_{c,j_{1}}S_{c,j_{2}}+\underset{i\in I,j\in J}{\sum }\gamma_{i,j}^{\text{V}}W_{c,i}S_{c,j}$ for all $w\in W^{\text{V}}\left(c,t\right)$ and $G_{c,w}=\gamma_{0,0}^{\text{R}}+\underset{i\in I}{\sum }\gamma_{0,i}^{\text{R}}W_{c,i,w}+\underset{i_{1}\leq i_{2}\in I}{\sum }\gamma_{i_{1},i_{2}}^{\text{R}}W_{c,i_{1},w}W_{c,i_{2},w}+\underset{j\in J}{\sum }\gamma_{0,j}^{\text{R}}S_{c,j}+\underset{j_{1}\leq j_{2}\in J}{\sum }\gamma_{j_{1},j_{2}}^{\text{R}}S_{c,j_{1}}S_{c,j_{2}}+\underset{i\in I,j\in J}{\sum }\gamma_{i,j}^{\text{R}}W_{c,i}S_{c,j}$ for all $w\in W^{\text{R}}\left(c,t\right)$.

For a given county *c* and year *t*, we dissect the crop yield into components G, E, and M as follows.

- Component E is defined as $\underset{w\in W\left(c,t\right)}{\sum }\max\left\{G_{c,w},0\right\}$, which is the maximally achievable growth potential determined by weather and soil variables and their interactions. The max function is used to narrow the range of summation of non-negative weekly growth potential terms during the favorable growing season.
- Component G is defined as $\overset{K}{\underset{k=0}{\sum }}\alpha_{c,k}t^{k}$. Year number *t* and parameter α are normalized so that component G is within [0, 1], indicating the proportion of component E that is achieved by genetics. This component captures what is not explained by environment and management variables, which is mostly due to genetic improvement over time. This component is estimated with a separate polynomial function for each crop reporting district.
- Component M is defined as $\frac{\underset{w\in W\left(c,t\right)}{\sum }D_{c,t}\left(P_{c,w}-H_{c,w}\right)G_{c,w}}{\underset{w\in W\left(c,t\right)}{\sum }\max\left\{G_{c,w},0\right\}}$, where parameters *D*, *P*, and *H* are all normalized to [0, 1]. As such, component M reflects the proportion of component E that is captured by management.

By definition, predicted yield is equal to the product of components G, E, and M. Notice that component E represents the maximally achievable growth potential from 1980 to 2019 when components G and M are normalized to [0, 1]. For future years when both seed genetics and management practices continue to improve, such growth potential may be exceeded.

### 3.4. Model Performance Evaluation

We used the following definition of the root mean square error (RMSE) to measure the prediction accuracy of the model:


(2) $$\begin{array}{l} r\left(\alpha ,\gamma ,CT\right)=\sqrt{\frac{\underset{\left(c,t\right)\in CT}{\sum }A_{c,t}^{2}{\left(y_{c,t}-{ŷ}_{c,t}\right)}^{2}}{\underset{\left(c,t\right)\in CT}{\sum }A_{c,t}^{2}}}. \end{array}$$

Here, parameters α and γ are used in equation (1) to calculate ŷ. Parameter *A*_*c,t*_, the area planted in county *c* and year *t*, is used as the weight.

The proposed model was evaluated based on both descriptive and predictive performances in the case study. The descriptive performance measures how well the model can fit the training data and explain the spatial and temporal variability of crop yield. Results from models (3)-(7) and (8)-(12) can provide insights on weather and soil interactions, trends of components of G, E, and M, and growth potential. The predictive performance evaluates the ability of the model to predict crop yield for unseen years or counties, for which training data have not been used to train the model. For this purpose, we trained the GEM model 40 times in a leave-one-year-out manner to validate its temporal prediction performance and 2,649 times in a leave-one-county-out manner to validate its spatial prediction performance. Moreover, the model was also used to provide in season prediction with daily updates of weather conditions.

Although numerous crop yield prediction models can be found in the literature, most were designed for different sets of explanatory variables and more focused geographic regions or time periods. To provide a meaningful benchmark comparison, we used the nearest-neighbor approach (Cover and Hart, 1967), which is popular for machine learning studies and intuitive in the crop yield prediction context. The nearest-neighbor approach for crop yield prediction was implemented as follows. To predict the yield of county *c* in an unseen year *t*, we identified historical yield data for county *c* in the nearest-year (before or after year *t*) and used that yield as the prediction. Similarly, to predict the yield of an unseen county *c* in year *t*, we identified historical yield data for the geographically nearest-county in the same year *t* and used that yield as the prediction. As such, the nearest-neighbor approach can be referred to more specifically as nearest-year and nearest-county for temporal and spatial predictions, respectively.

### 3.5. Algorithm

The GEM model (1) is a complex nonlinear optimization problem that is not readily solvable by standard machine learning algorithms. Herein, we present a heuristic algorithm that can efficiently obtain a high quality solution (without optimality guarantee) for unknown parameters α and γ. The strategy is not to simultaneously optimize α and γ but to iteratively update one of them at a time while keeping the other fixed. As such, solving model (1) reduces to solving two smaller quadratic optimization models multiple times, which are readily solvable by state-of-the-art optimization solvers such as Gurobi (Gurobi Optimization, LLC, 2021) and Cplex (IBM ILOG Cplex, 2009). Detailed steps of the algorithm are explained as follows.

**Step 0: Initialization**. Pre-process data by normalizing them to [0, 1]. Initialize the incumbent α^*^ as $\alpha_{c,0}^{*}=1,\forall c\in C$, $\alpha_{c,k}^{*}=0,\forall c\in C,k\in \left\{1,...,K\right\}$, and $\gamma_{i,j}^{*}=0,\forall i,j$. Go to step 1.

Step 1: Update γ^*^. Randomly select a subset ${CT}^{1}\subset CT$ with approximately 80% samples. Solve the following quadratic optimization model using ${CT}^{1}$ while keeping the incumbent α^*^ as a constant.


(3) $$\begin{array}{ll} \underset{\gamma ,G,ŷ}{\max} & \underset{\left(c,t\right)\in {CT}^{1}}{\sum }A_{c,t}^{2}{\left(y_{c,t}-{ŷ}_{c,t}\right)}^{2} \end{array}$$

(4) $$\begin{array}{c} s.t.\;{ŷ}_{c,t}=\left(\overset{K}{\underset{k=0}{\sum }}\alpha_{c,k}^{*}t^{k}\right)\cdot \left[\underset{w\in W\left(c,t\right)}{\sum }D_{c,t}\left(P_{c,w}-H_{c,w}\right)G_{c,w}\right]\\ \forall \left(c,t\right)\in {CT}^{1} \end{array}$$

(5) $$\begin{array}{l} G_{c,w}=\gamma_{0,0}^{\text{V}}+\underset{i\in I}{\sum }\gamma_{0,i}^{\text{V}}W_{c,i,w}+\underset{i_{1}\leq i_{2}\in I}{\sum }\gamma_{i_{1},i_{2}}^{\text{V}}W_{c,i_{1},w}W_{c,i_{2},w}\\ \;+\underset{j\in J}{\sum }\gamma_{0,j}^{\text{V}}S_{c,j}+\underset{j_{1}\leq j_{2}\in J}{\sum }\gamma_{j_{1},j_{2}}^{\text{V}}S_{c,j_{1}}S_{c,j_{2}}\\ \;+\underset{i\in I,j\in J}{\sum }\gamma_{i,j}^{\text{V}}W_{c,i}S_{c,j}\;\forall w\in W^{\text{V}}\left(c,t\right) \end{array}$$

(6) $$\begin{array}{l} G_{c,w}=\gamma_{0,0}^{\text{R}}+\underset{i\in I}{\sum }\gamma_{0,i}^{\text{R}}W_{c,i,w}+\underset{i_{1}\leq i_{2}\in I}{\sum }\gamma_{i_{1},i_{2}}^{\text{R}}W_{c,i_{1},w}W_{c,i_{2},w}\\ \;+\underset{j\in J}{\sum }\gamma_{0,j}^{\text{R}}S_{c,j}+\underset{j_{1}\leq j_{2}\in J}{\sum }\gamma_{j_{1},j_{2}}^{\text{R}}S_{c,j_{1}}S_{c,j_{2}}\\ \;+\underset{i\in I,j\in J}{\sum }\gamma_{i,j}^{\text{R}}W_{c,i}S_{c,j}\;\forall w\in W^{\text{R}}\left(c,t\right) \end{array}$$

(7) $$\begin{array}{l} 0\leq {ŷ}_{c,t}\leq 300\;\forall \left(c,t\right)\in {CT}^{1} \end{array}$$

Let $\tilde{\gamma }$ denote an optimal solution and use it to define a new incumbent candidate as ${\tilde{\gamma }}^{*}=0.2\gamma^{*}+0.8\tilde{\gamma }$. If $r\left(\alpha^{*},{\tilde{\gamma }}^{*},CT\right)<r\left(\alpha^{*},\gamma^{*},CT\right)$, then update $\gamma^{*}\leftarrow {\tilde{\gamma }}^{*}$. Go to step 2.

Step 2: Update α^*^. Randomly select a subset ${CT}^{2}\subset CT$ with approximately 80% samples. Solve the following quadratic optimization model using ${CT}^{2}$ while keeping G^*^ determined by the incumbent γ^*^ as a constant.


(8) $$\begin{array}{ll} \underset{\alpha ,ŷ}{\max} & \underset{\left(c,t\right)\in {CT}^{2}}{\sum }A_{c,t}^{2}{\left(y_{c,t}-{ŷ}_{c,t}\right)}^{2} \end{array}$$

(9) $$\begin{array}{c} s.t.\;{ŷ}_{c,t}=\left(\overset{K}{\underset{k=0}{\sum }}\alpha_{c,k}t^{k}\right)\cdot \left[\underset{w\in W\left(c,t\right)}{\sum }D_{c,t}\left(P_{c,w}-H_{c,w}\right)G_{c,w}^{*}\right]\\ \forall \left(c,t\right)\in {CT}^{2} \end{array}$$

(10) $$\begin{array}{c} \alpha_{c,k}=\alpha_{d,k}\\ \forall d\in R\left(c\right),k\in \left\{0,...,K\right\} \end{array}$$

(11) $$\begin{array}{c} 0\%\leq \overset{K}{\underset{k=0}{\sum }}\alpha_{c,k}t^{k}\leq 100\%\\ \forall \left(c,t\right)\in {CT}^{2} \end{array}$$

(12) $$\begin{array}{c} -2.5\%\leq \overset{K}{\underset{k=0}{\sum }}\alpha_{c,k}\left[t^{k}-{\left(t-1\right)}^{k}\right]\leq 5\%\\ \forall \left(c,t\right)\in {CT}^{2} \end{array}$$

Let $\tilde{\alpha }$ denote an optimal solution and use it to define a new incumbent candidate as ${\tilde{\alpha }}^{*}=0.2\alpha^{*}+0.8\tilde{\alpha }$. If $r\left({\tilde{\alpha }}^{*},\gamma^{*},CT\right)<r\left(\alpha^{*},\gamma^{*},CT\right)$, then update $\alpha^{*}\leftarrow {\tilde{\alpha }}^{*}$.

Terminate the algorithm if the incumbent solution (α^*^, γ^*^) has not been updated for two consecutive iterations; otherwise go back to Step 1 for a new iteration.

**Remark for model (3)-(7):** Minimizing the objective function (3) is equivalent to minimizing the RMSE since the square root function is a monotonically increasing one. With parameter α^*^ being a constant, all constraints (4)-(7) are linear. Constraint (7) avoids the predicted yield to be negative or unrealistically high.

**Remark for model (8)-(12):** Constraint (10) requires that all α_*c,k*_ values in a same crop reporting district be the same. Constraint (11) normalizes the genetic progress within [0%, 100%]. Constraint (12) restricts the change in genetic performance over the previous year to be between −2.5% and 5%. These lower and upper bounds were subjectively estimated to reflect changes in genetic perform, and the optimal γ was found to be insensitive to these parameters.

**Remark for incumbent updates:** The incumbent solution (α^*^, γ^*^) is not automatically updated with optimal solution $\left(\tilde{\alpha },\tilde{\gamma }\right)$ from the two quadratic optimization models. Rather, it is only partially updated by $\left(\tilde{\alpha },\tilde{\gamma }\right)$ if the new incumbent candidate passes a cross validation. In particular, the optimal solutions $\tilde{\alpha }$ and $\tilde{\gamma }$ are obtained using random subsets of CT, then new incumbent candidate solutions are defined as ${\tilde{\gamma }}^{*}=0.2\gamma^{*}+0.8\tilde{\gamma }$ and ${\tilde{\alpha }}^{*}=0.2\alpha^{*}+0.8\tilde{\alpha }$, which will not be accepted unless they improve the RMSE on the entire data set CT. As such, this technique reduces overfitting by cross validating the generalizability of any updates made to the incumbent solution. Performance of the algorithm was found to be insensitive to the parameters 0.2 and 0.8.

## 4. Results and Discussions

Computational experiments were carried out using Matlab as the main platform and Gurobi 9.1 as the quadratic programming solver. The proposed heuristic algorithm took approximately 10 min to find a high quality solution on an average laptop. Data and Matlab code used in this study were shared at https://github.com/lzwang2017/maizeyield. Section 4.1 gives the results of fitting the entire data set and section 4.2 presents the results of predicting for unseen years or counties.

### 4.1. Descriptive Performance

#### 4.1.1. Training Error

**Figure 10:**
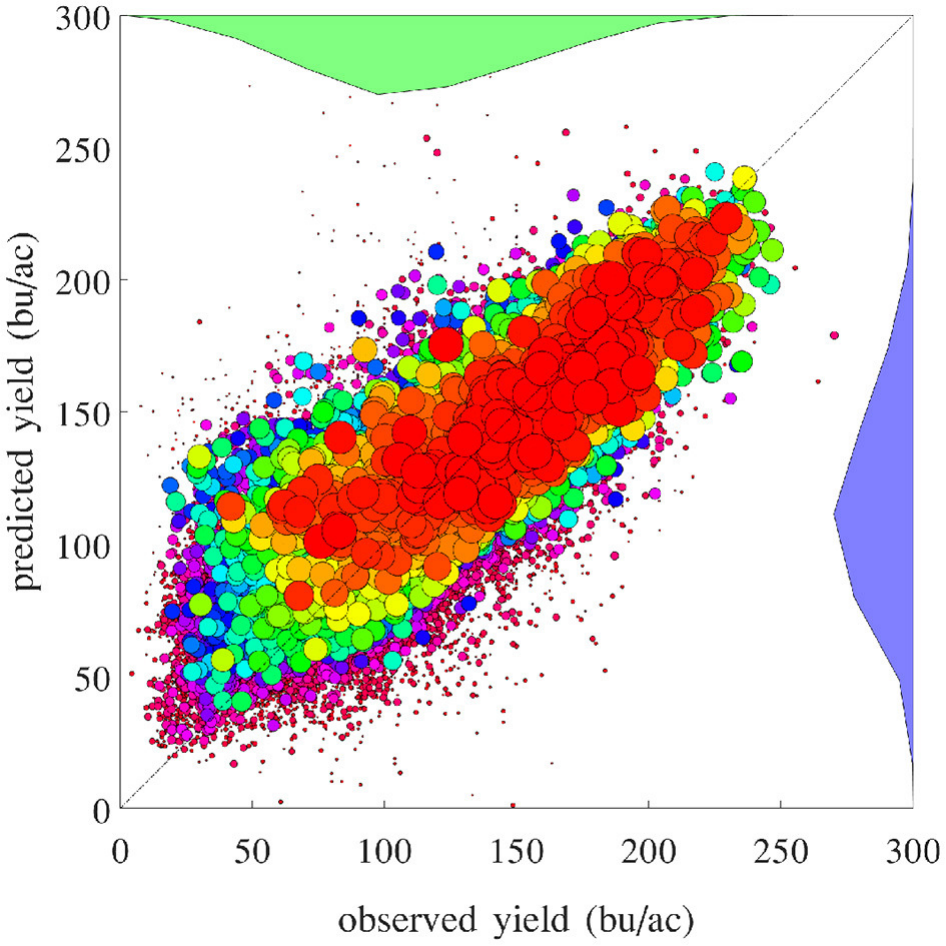
Predicted vs. observed corn yield for all 78,169 county-year combinations are plotted against the 45 degree line, with the sizes of the dots approximately proportional to the areas planted. Histograms of the two sets of yield are also shown.

**Figure 11:**
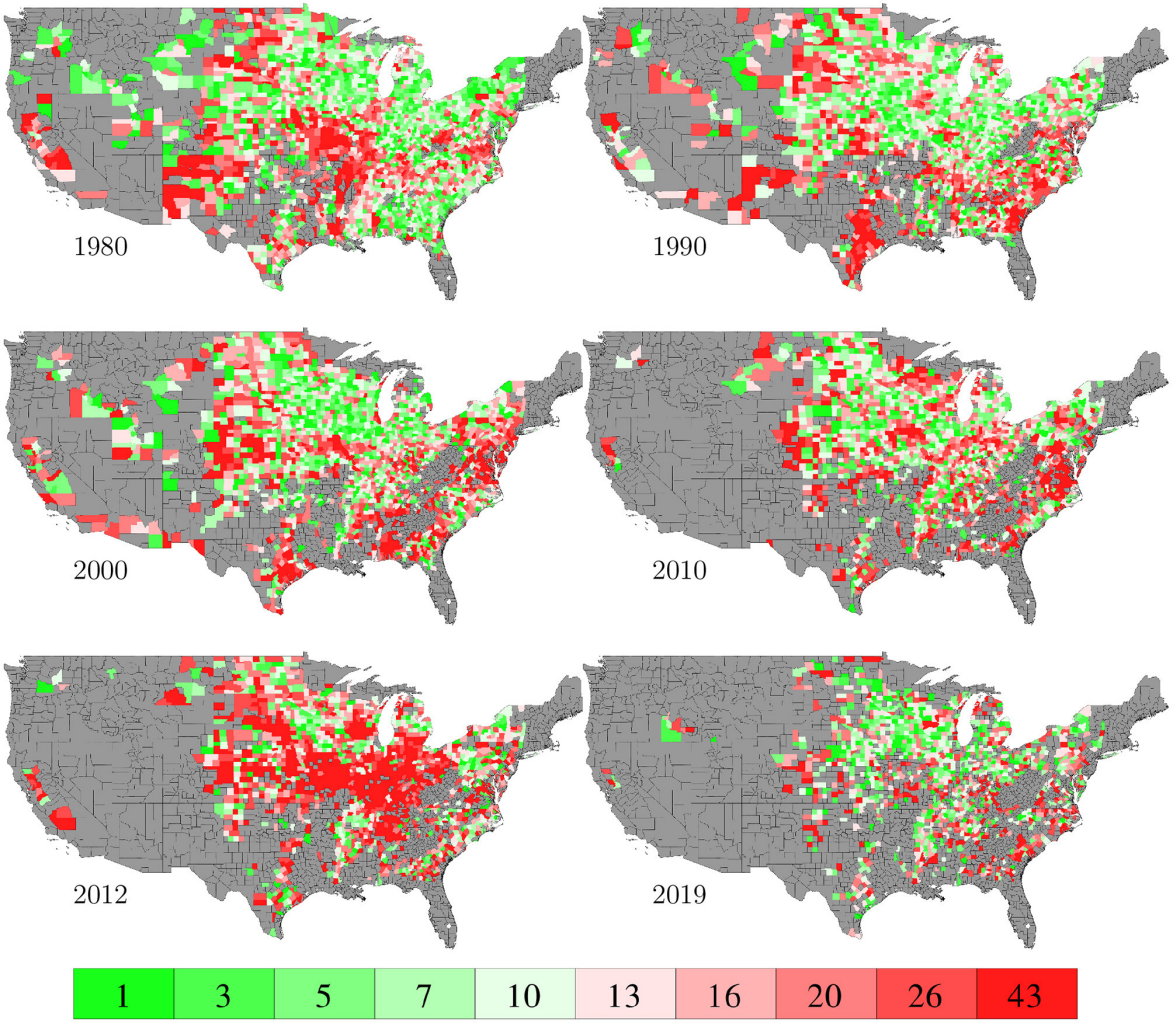
Training RMSEs. The color map shows the averages of the 0–10th, 10–20th,…, 90–100th percentile intervals of all county-year combinations, weighted by the areas planted. Counties without yield data are shown in gray.

The RMSE of fitting the entire data set with the GEM model is 17.84 bu/ac, which is 10.34% of the average yield in 2019. Figure 10 plots the predicted and observed yield for all 78,169 county-year combinations against the 45 degree line; it also compares the histograms of the two sets of yields. Figure 11 visualizes the spatial variability of training RMSEs in 6 representative years. In order to offset the potentially misleading discrepancy between the geographic area of a county shown on the map and the area planted in the county, we designed the color map in such a way that each color represents 10 percent of total areas planted. Similarly designed color maps are also used in other figures. Figure 11 suggests that more than 70% of historical yield data were explained by the GEM model within a 10% relative error; counties with larger planted areas had lower errors than those with smaller planted areas.

#### 4.1.2. Weather and Soil Interactions

**Figure 12:**
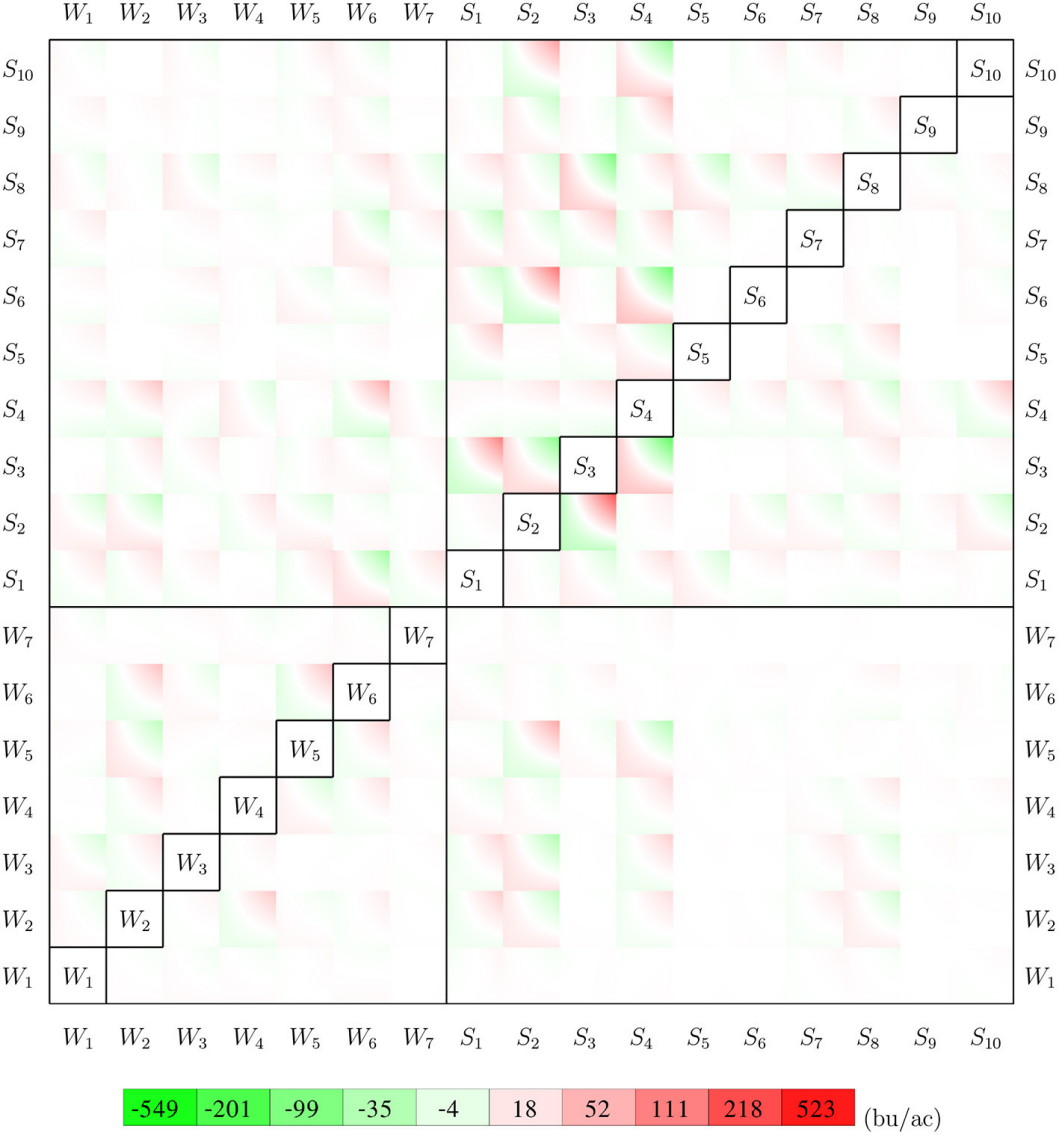
Interactions between 7 weather variables and 10 soil variables. The top left side of the diagonal shows the interactive effects for the vegetative stage, and the bottom right is for the reproductive stage. All weather and soil variables are normalized to [0, 1].

Parameter γ from the GEM model reveals how weather and soil variables jointly determine the growth potential in the vegetative and reproductive stages. Figure 12 visualizes such pair-wise interactive effects with a color map. The color square for variables *i* and *j* shows the combined effects of $\gamma_{i,j}+\frac{1}{16}\left(\gamma_{i,0}+\gamma_{i,i}+\gamma_{0,j}+\gamma_{j,j}\right)$. For a given set of observations for 7 weather variables and 10 soil variables of a given week, the growth potential for that week can be calculated using information from Figure 12 as follows. First, locate the 272 intersections with the 17 variables in vertical and horizontal directions less the diagonal. Then determine if the crop is in vegetative or reproductive stage in the given week. Finally, the growth potential for the vegetative or reproductive stage can be calculated as the summation of the 136 values at the intersections inside the 136 squares on the top left, or bottom right, side of the diagonal, respectively. The asymmetry in the figure reveals how maize may respond differently to the same environmental conditions during the vegetative and reproductive stages.

Results from Figure 12 can be used to determine the sensitivity of crop yield to combinations of specific weather and/or soil variables. For example, the (*S*_4_, *W*_6_) square suggests that the combination of higher tks and higher tmin in the vegetative stage had an unfavorable effect on crop yield, but the effect of the same combination was negligible in the reproductive stage, as suggested by the (*W*_6_, *S*_4_) square.

#### 4.1.3. Trends of Components G, E, and M

**Figure 13:**
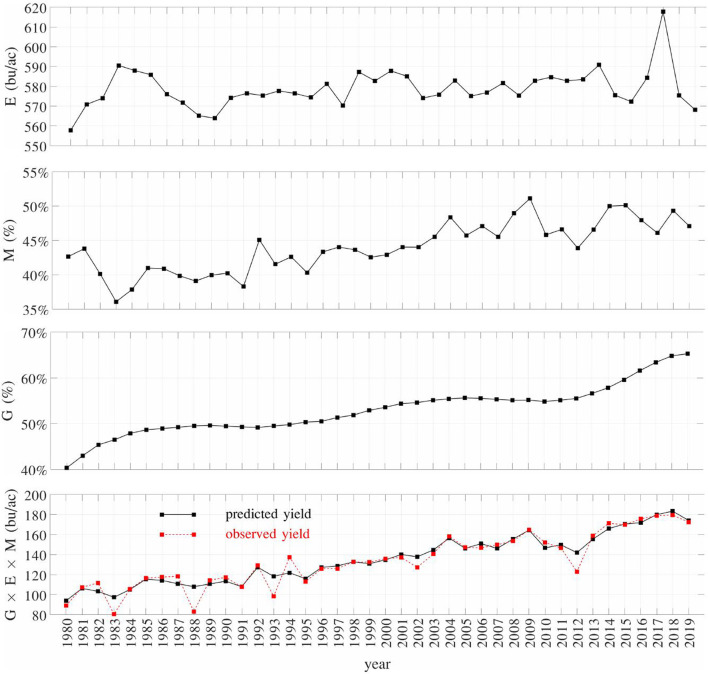
Trend of national averages of G, E, M from 1980 to 2019. The predicted yield is the product of G, E, and M.

Figure 13 shows the trends of the average components G, E, and M defined in section 3.3, as well as predicted and observed yield from 1980 to 2019. Component E shows an average growth potential of 576 bu/ac, which fluctuates from year to year with a slight increasing trend in the long term and a sharp decrease in recent years. This result is consistent with recent observations by meteorologist Takle and atmospheric scientist Gutowski (Takle and Gutowski, 2020). Both components M and G demonstrate clear increasing trends; the former reflects the improvement in population density and planting/harvesting timing, whereas the latter explains the increasing trend of yield unaccounted for by components E and M. The product of components G, E, and M accurately fits the observed yield except for several years with extreme weathers (e.g., 1983, 1988, 1993, and 2012). This may be caused by the lack of sufficient data with extreme weathers for the model to learn how maize responds to stressful environmental conditions. Improving prediction accuracy under extreme weather conditions has been widely recognized as a challenging yet important topic for future research (van der Velde et al., 2012; Eitzinger et al., 2013; Blanc, 2017; Schauberger et al., 2017), especially in the face of global climate changes.

#### 4.1.4. Growth Potential

**Figure 14:**
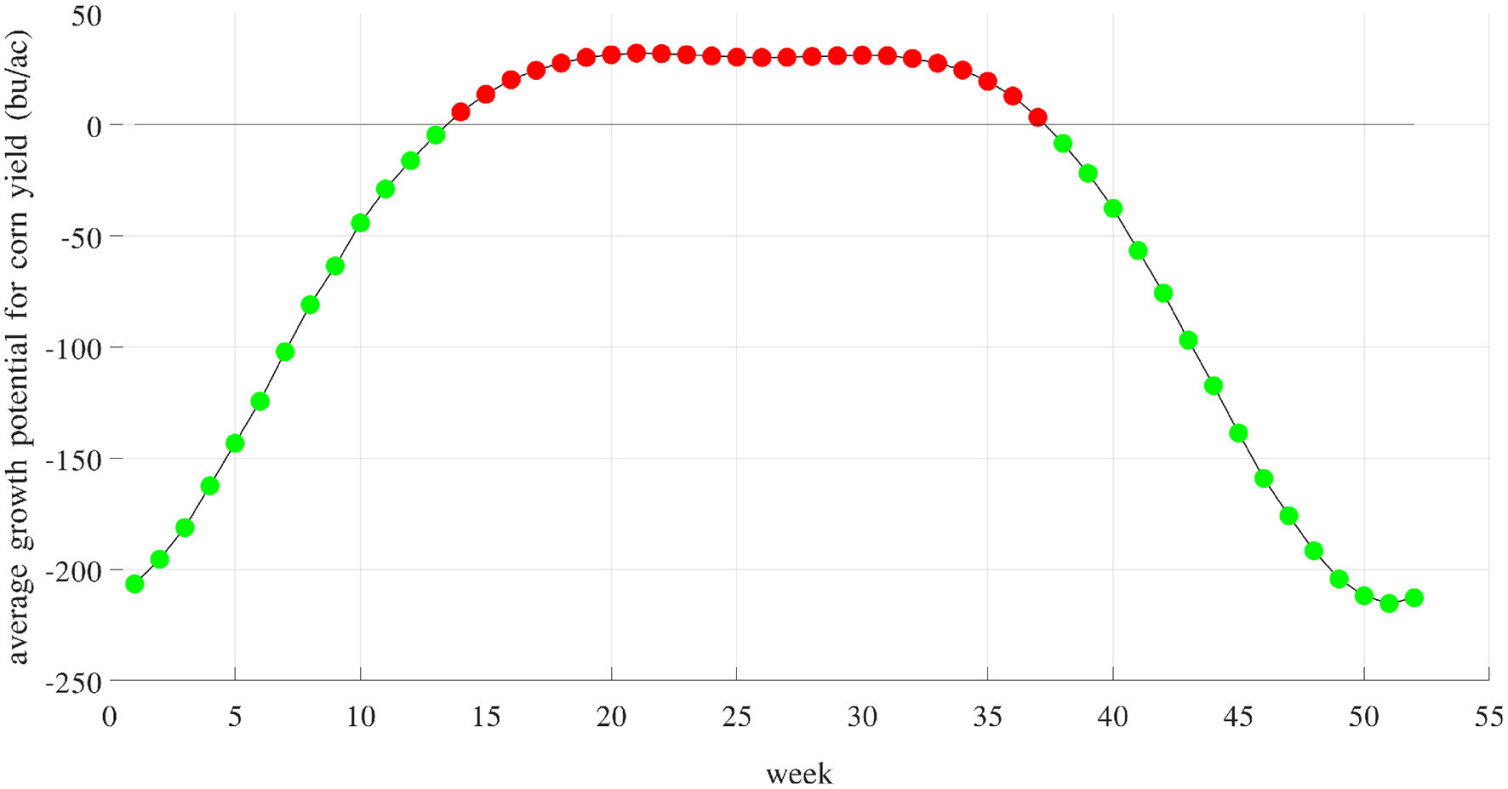
Weekly growth potential determined by weather and soil variables and their interactions. Red and green colors indicate positive and negative effects, respectively. These potentials are positive from week 14 (late March) to week 37 (early September).

**Figure 15:**
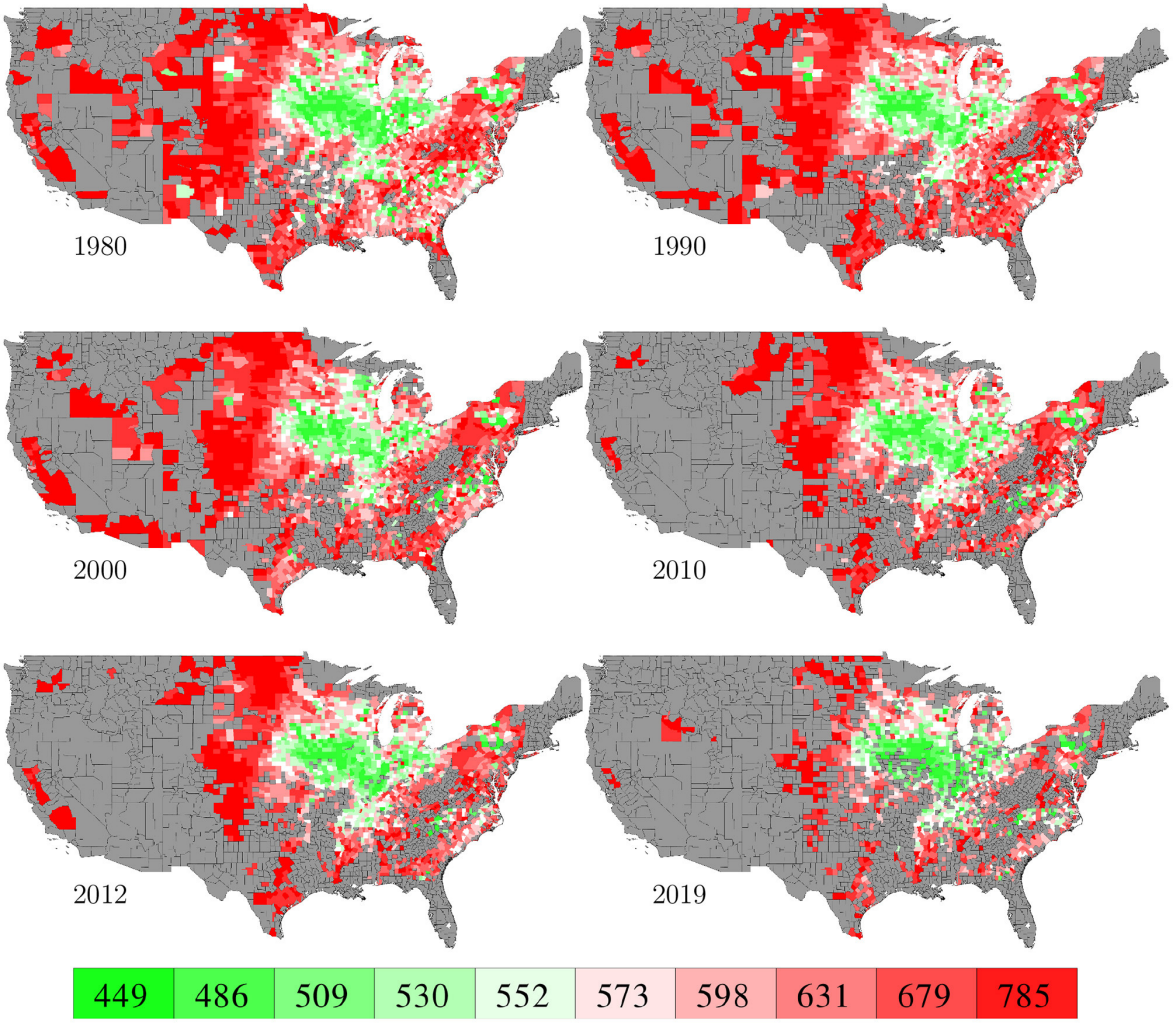
Growth potential determined by weather and soil in 6 representative years. The color map shows the averages of the 0–10th, 10–20th,…, 90–100th percentile intervals of all county-year combinations, weighted by areas planted. Counties without yield data are shown in gray.

Figure 14 shows the average component E for 52 weeks of the year. Averaged across 2,649 counties and 40 years, these growth potentials are positive from week 14 (late March) to week 37 (early September), indicating a favorable time window for maize growth. These results are consistent with prior work that documented the yield benefits of earlier planting and longer season varieties (Kucharik, 2006; Zhu et al., 2018). Figure 15 visualizes the spatial variability of growth potential in 6 representative years, ranging from 456 to 714 bu/ac. As a reality check, according to the National Corn Growers Association (NCGA, 2020), the winners of the National Corn Yield Contest in 2019 and 2020 achieved, respectively, 616 (new record) and 476 bushels per acre.

### 4.2. Predictive Performance

#### 4.2.1. Temporal Prediction

Forty experiments were conducted, for 1 year at a time, to test how accurately the GEM model can be used to predict the yield of an unseen year for which historical yield were held out of the training data. Complete weather data for the target year were provided to the prediction model. Section 4.2.3 presents results for in season prediction with daily updated weather information. These forty experiments took approximately seven CPU hours.

**Figure 16:**
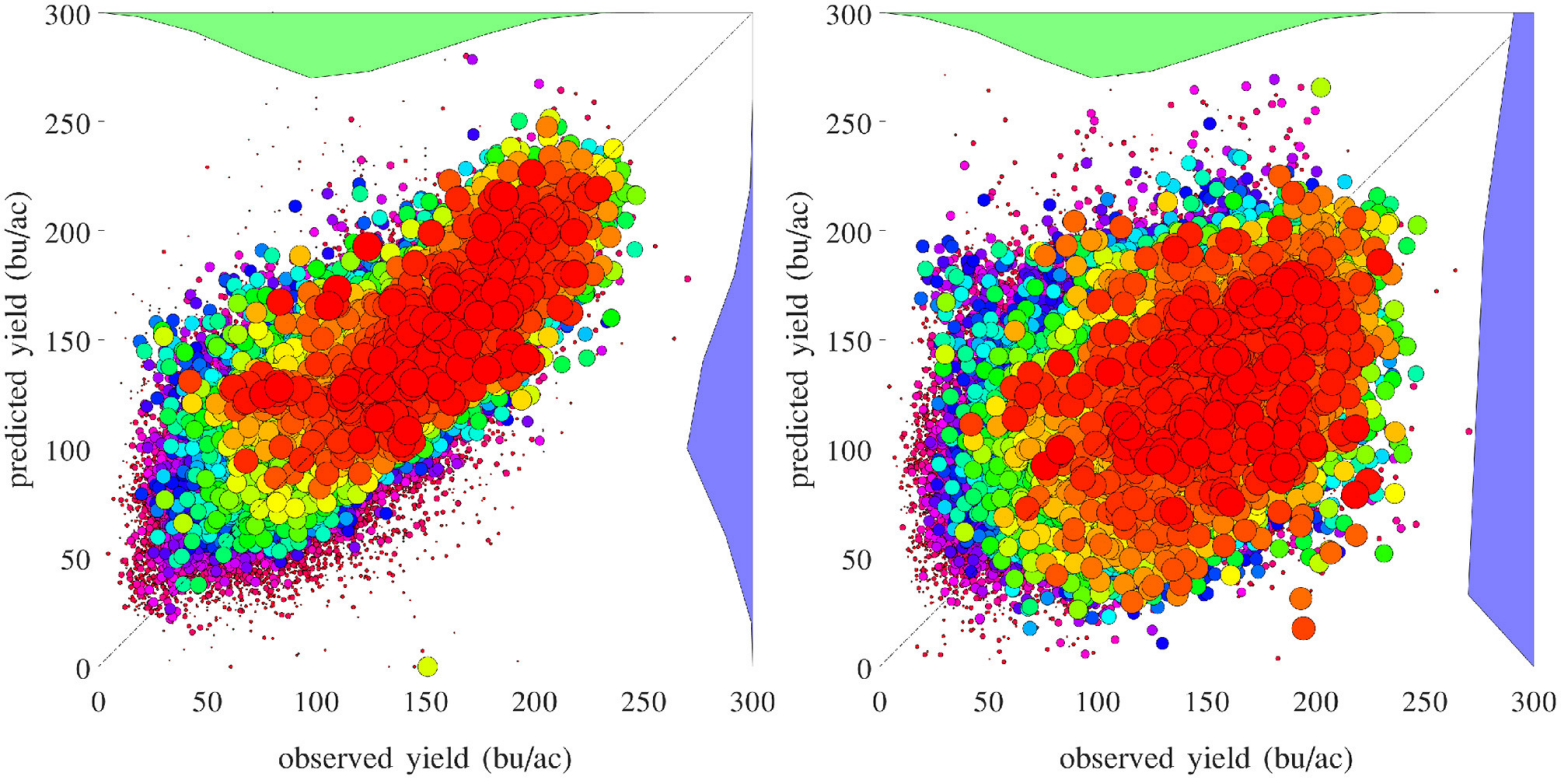
Performances of temporal prediction of the GEM model (left) and nearest-year approach (right). Predicted vs. observed corn yield for all 78,169 county-year combinations are plotted against the 45 degree line, with the sizes of the dots approximately proportional to the areas planted. Histograms of the two sets of yield are also shown.

**Figure 17:**
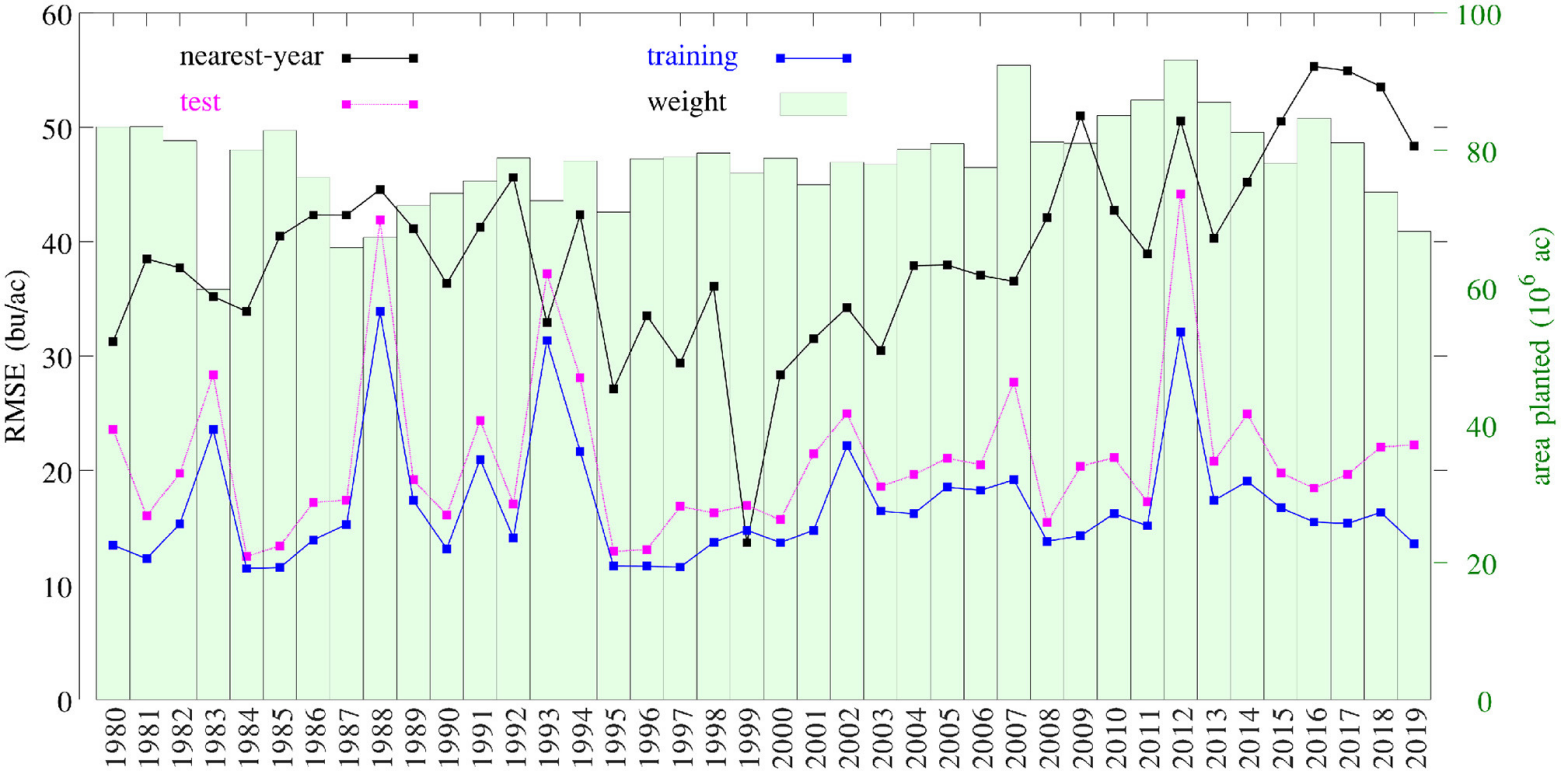
RMSEs of training, test, and nearest-year models. Total planted areas were shown to illustrate the weight for each year.

Figure 16 compares the performances of temporal prediction of the GEM model and nearest-year approach, in which predicted and observed yields for all 78,169 county-year combinations are plotted against the 45 degree line. The RMSEs for these two models were 22.25 and 40.66 bu/ac, respectively. The weighted *R*^2^ values for these two models were 0.79 and 0.30 bu/ac, respectively. Figure 17 compares the RMSEs for training (using full data), test (leaving 1 year out at a time) using the GEM model, and test using the nearest-year approach from 1980 to 2019. This figure shows that the GEM model clearly outperformed the nearest-year approach. Moreover, the small gap between training and testing errors also indicates very little over-fitting in the model, thanks to the integration of domain knowledge in the design of the model as well as the large data set that allows the model to extract temporally and spatially transferable information. These results suggested that the proposed GEM model can be used to produce crop yield predictions of a known county (with training data) for a new year based on its soil, weather, and management variables.

#### 4.2.2. Spatial Prediction

**Figure 18:**
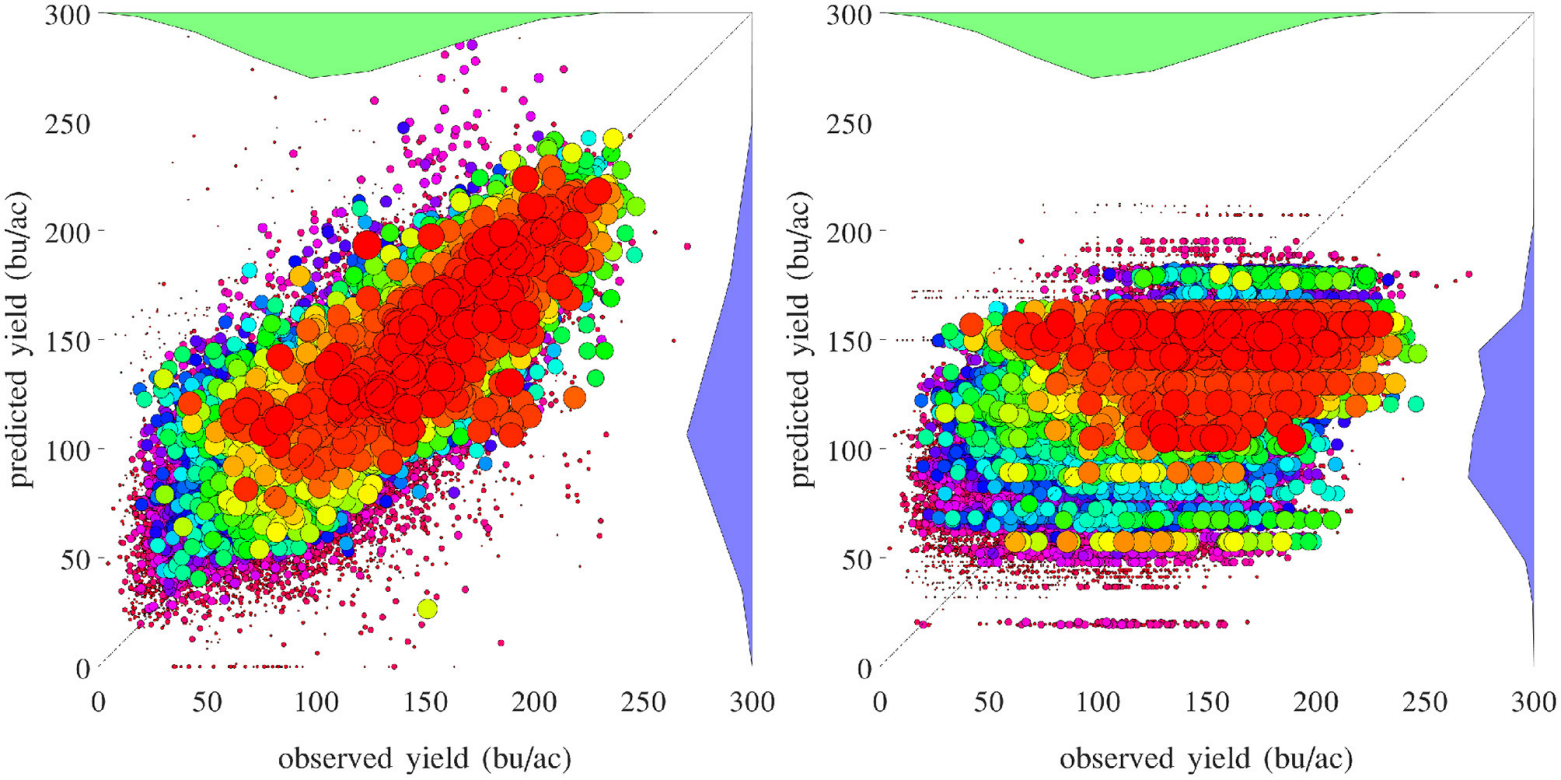
Performances of spatial prediction of the GEM model (left) and nearest-county approach (right). Predicted vs. observed corn yield for all 78,169 county-year combinations are plotted against the 45 degree line, with the sizes of the dots approximately proportional to the areas planted. Histograms of the two sets of yield are also shown.

**Figure 19:**
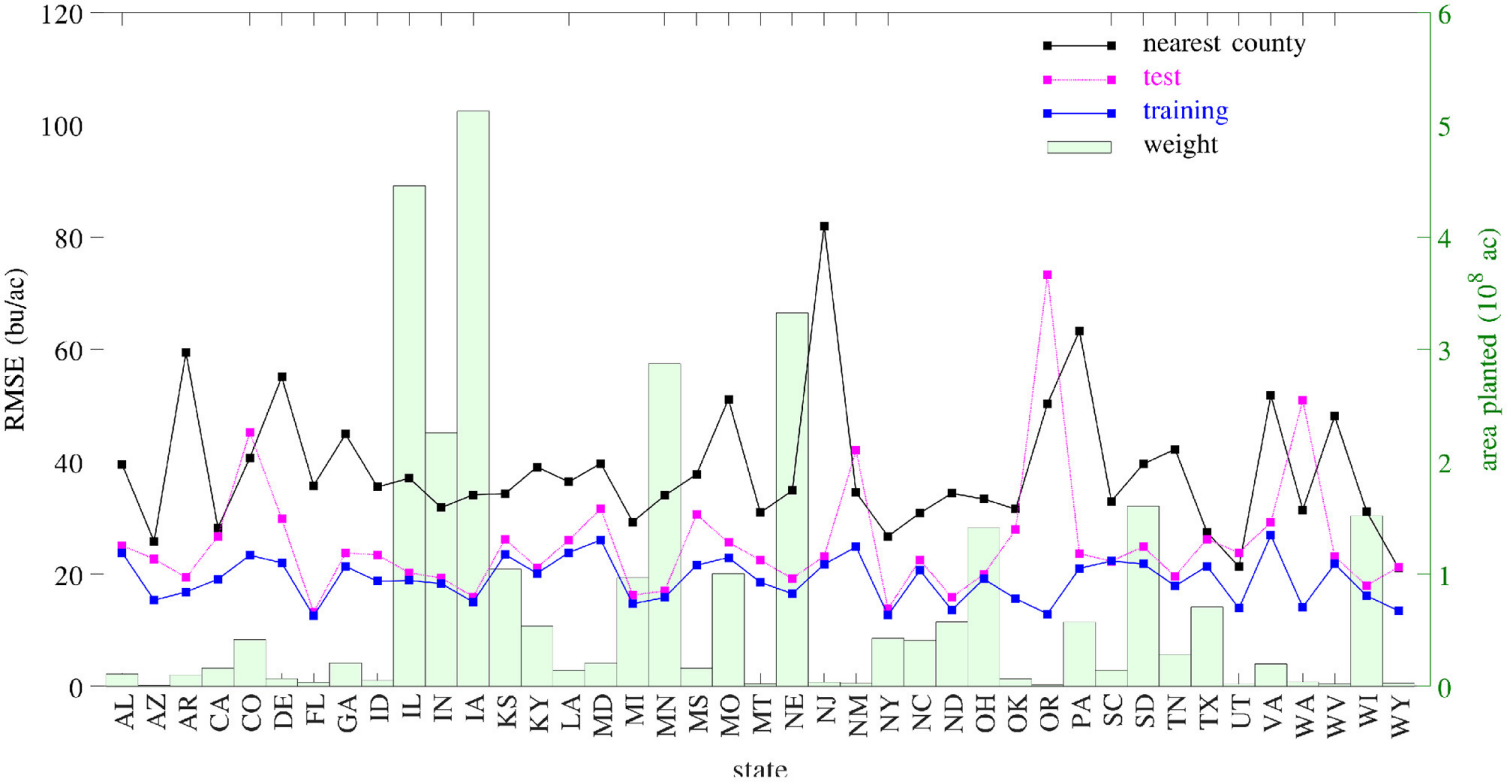
RMSEs of training, test, and nearest-county models. Total planted areas were shown to illustrate the weight for each state.

A set of 2,649 experiments were conducted, for one county at a time, to test how accurately the GEM model can be used to predict the yield of an unseen county for which historical yield were held out of the training data. Complete weather data for all years were provided to the prediction model. These experiments took approximately 18 CPU days. Figure 18 compares the performances of spatial prediction of the GEM model and nearest-neighbor approach, in which predicted and observed yields for all 78,169 county-year combinations are plotted against the 45 degree line. The RMSEs for these two models were 19.97 and 35.67 bu/ac, respectively. The weighted *R*^2^ values for these two models were 0.83 and 0.46 bu/ac, respectively. Figure 19 compares the RMSEs for training (using full data), test (leaving one county out at a time) using the GEM model, and test using the nearest-county approach for 41 states. The GEM model demonstrated superior performance over the nearest-county approach and very little overfitting. Therefore, the GEM model can be used to produce crop yield predictions for a new county based on its soil, weather, and management variables.

#### 4.2.3. In Season Prediction With Daily Updates

**Figure 20:**
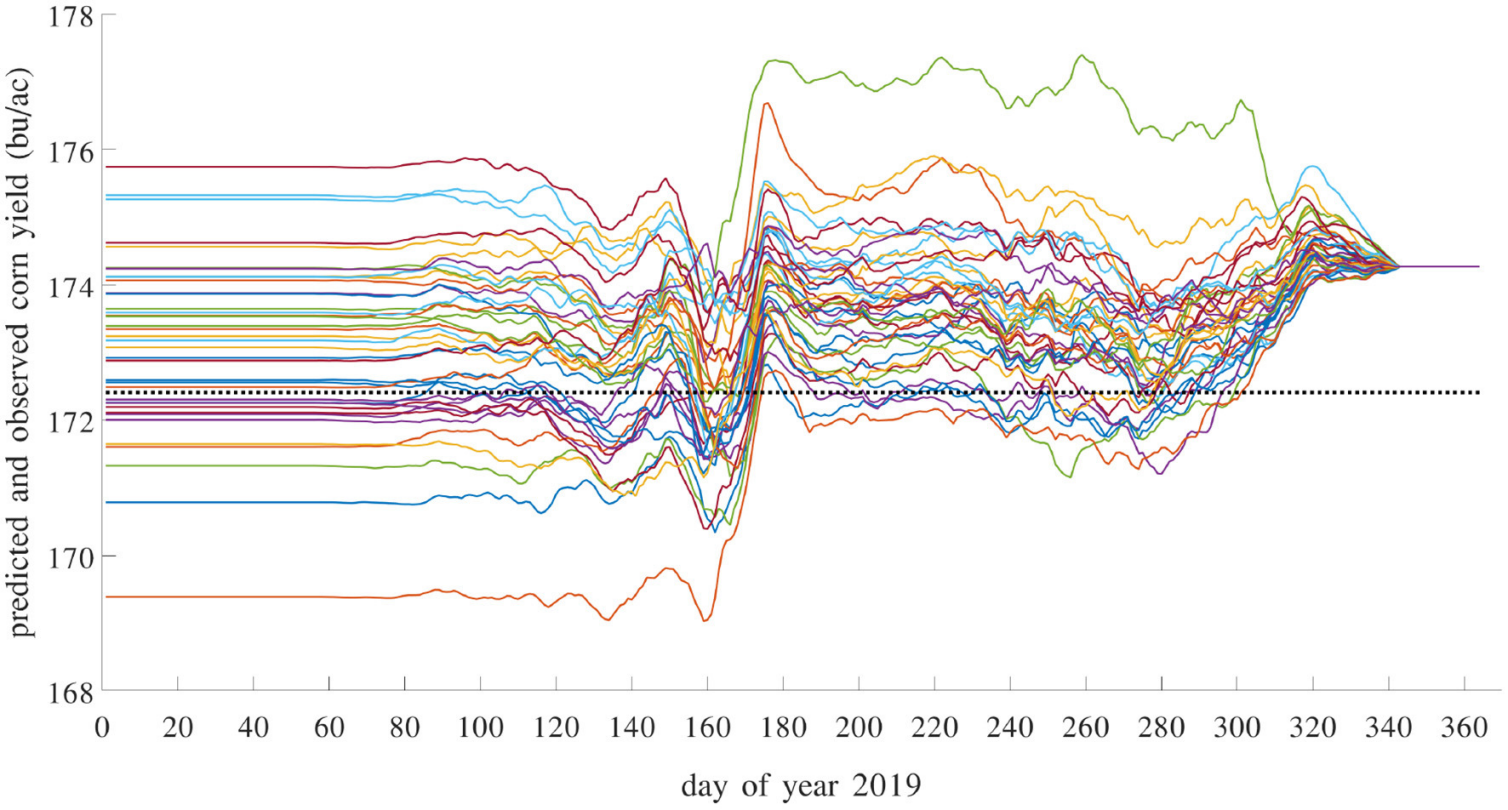
Predicted and observed average yield in 2019 with daily updates. Prediction model was trained using data from 1980 to 2018. There are 39 solid curves, representing 39 predictions using historical weather scenarios from 1980 to 2018, each giving a daily updated prediction using weather data from a historical year to impute the unobserved weather variables in 2019. The dashed flat line is the observed average yield over 1,256 counties with yield data for 2019.

Most existing methods for in season yield prediction use remote sensing data (Teal et al., 2006; Jagmandeep et al., 2020). The proposed GEM model can be used to provide daily updated yield predictions using daily updated weather data. We demonstrated this approach for the test year 2019 using a GEM model trained with data from 1980 to 2018. For each day in 2019, we made a yield prediction by combining the observed weather data (from January 1st to that day) in 2019 with unobserved weather data (from the next day to December 31st) from a historical year. As such, 39 different predictions were made using 39 historical weather scenarios from 1980 to 2018. These predictions differ widely on day 1 and then gradually converge as more actual weather information in 2019 has been observed. These results are shown in Figure 20. Similar daily updated crop yield predictions can be produced using the proposed GEM model for any known county with specific soil and management conditions as long as daily weather variables are available.

## 5. Conclusions

Crop yield is a complex trait jointly determined by numerous genotype, environment, and management variables and their interactions. Being able to accurately predict crop yield under changing environmental conditions in a wide range of geographic locations is increasingly important to agriculture stakeholders. It also poses a formidable academic challenge, which can only be overcome by integrating insights from crop science with data analytical methodology.

In our attempt to explain the temporal and spatial variability of maize yield in the U.S., we collected a large data set and designed the GEM model to analyze the data. The data covers 40 years of yield, weather, soil, and management information from 41 states. The GEM model was specifically designed for this data set to extract insights that are explainable and transferable both spatially and temporally.

Computational results suggest that the GEM model is a reasonable attempt to combine the strengths of data driven models in prediction accuracy and the advantage of knowledge driven models in explainability. Compared with data driven models in the literature, the GEM model achieved a prediction accuracy on par with state-of-the-art machine learning models, thus the advantage of the GEM model is the explainable insights. For example, predicted yield is dissected into genetics, environment, and management components. Compared with knowledge driven models in the literature, the GEM model has a more flexible modeling structure that allows unknown parameters to be efficiently and optimally calibrated using advanced computational techniques, extracting more data-driven information and less human biases.

The GEM model has several limitations and caveats. First, the model was specifically designed for the current data set and not directly applicable to other data sets without necessary modifications, although similar modeling and solution techniques will still be applicable. Second, several assumptions listed in section 3.1 are the backbone of the model, which allow the model to be constructed and solved efficiently but may also limit the effectiveness of the model to a certain extent. Third, several important variables (such as seed genotype, irrigation, fertilization, tillage, and disease/weed control) were not included in the model due to lack of public data sources at the desired scale.

Several followup research directions deserve further investigation. Similar modeling and solution techniques can be applied to other crops, other regions, other time periods, and other data sets. More crop growth stages can be considered to incorporate more crop physiological insights and to give the GEM model additional features. Further effort should also be made to train the model to learn from low frequency but high impact extreme weather scenarios.

## Data Availability Statement

The original contributions generated for the study are included in the article/supplementary material, further inquiries can be directed to the corresponding author/s.

## Author Contributions

The author confirms being the sole contributor of this work and has approved it for publication.

## Funding

This work was partially supported by NSF under LEAP HI and GOALI programs (grant no. 1830478) and EAGER program (grant no. 1842097) and by the Plant Sciences Institute at Iowa State University.

## Author Disclaimer

Any opinions, findings, conclusions, or recommendations expressed in this publication are those of the author and do not necessarily reflect the view of the funding agencies.

## Conflict of Interest

The author declares that the research was conducted in the absence of any commercial or financial relationships that could be construed as a potential conflict of interest.

## Publisher's Note

All claims expressed in this article are solely those of the authors and do not necessarily represent those of their affiliated organizations, or those of the publisher, the editors and the reviewers. Any product that may be evaluated in this article, or claim that may be made by its manufacturer, is not guaranteed or endorsed by the publisher.
